# Involvement of l-Cysteine Desulfhydrase and Hydrogen Sulfide in Glutathione-Induced Tolerance to Salinity by Accelerating Ascorbate-Glutathione Cycle and Glyoxalase System in *Capsicum*

**DOI:** 10.3390/antiox9070603

**Published:** 2020-07-10

**Authors:** Cengiz Kaya, Bernardo Murillo-Amador, Muhammad Ashraf

**Affiliations:** 1 Soil Science and Plant Nutrition Department, Agriculture Faculty, Harran University, Sanliurfa 6300, Turkey; 2 Centro de Investigaciones Biológicas del Noroeste, S.C. Avenida Instituto Politécnico Nacional No. 195, Colonia Playa Palo de Santa Rita Sur, La Paz 23096, Baja California Sur, Mexico; 3 Department of Botany, University of Agriculture, Faisalabad 38040, Pakistan; vc@uaf.edu.pk

**Keywords:** oxidative stress, salinity tolerance, nonenzymatic metabolites

## Abstract

The aim of this study is to assess the role of l-cysteine desulfhydrase (l-DES) and endogenous hydrogen sulfide (H_2_S) in glutathione (GSH)-induced tolerance to salinity stress (SS) in sweet pepper (*Capsicum annuum* L.). Two weeks after germination, before initiating SS, half of the pepper seedlings were retained for 12 h in a liquid solution containing H_2_S scavenger, hypotaurine (HT), or the l-DES inhibitor dl-propargylglycine (PAG). The seedlings were then exposed for three weeks to control or SS (100 mmol L^−1^ NaCl) and supplemented with or without GSH or GSH+NaHS (sodium hydrosulfide, H_2_S donor). Salinity suppressed dry biomass, leaf water potential, chlorophyll contents, maximum quantum efficiency, ascorbate, and the activities of dehydroascorbate reductase, monodehydroascorbate reductase, and glyoxalase II in plants. Contrarily, it enhanced the accumulation of hydrogen peroxide, malondialdehyde, methylglyoxal, electrolyte leakage, proline, GSH, the activities of glutathione reductase, peroxidase, catalase, superoxide dismutase, ascorbate peroxidase, glyoxalase I, and l-DES, as well as endogenous H_2_S content. Salinity enhanced leaf Na^+^ but reduced K^+^; however, the reverse was true with GSH application. Overall, the treatments, GSH and GSH+NaHS, effectively reversed the oxidative stress and upregulated salt tolerance in pepper plants by controlling the activities of the AsA-GSH and glyoxalase-system-related enzymes as well as the levels of osmolytes.

## 1. Introduction

Sweet pepper is an important vegetable crop, especially in the Mediterranean region. It is usually grown in greenhouses to obtain high yields and good quality fruit in comparison with that from field conditions [1]. It is believed that sweet pepper can moderately tolerate saline stress, which is ascribed to reduced plant growth and yield [2,3].

Salinized soil and saltwater are the main reasons for the reduced production of most crops [4,5]. Salt stress causes a buildup of sodium (Na^+^) and chloride (Cl^−^), which causes osmotic and ionic stresses in plants [6,7]. This leads to detrimental effects on both metabolic and biochemical events in plants, and thus causes the inhibition of plant growth and development [8]. Salinity also decreases the uptake of water and essential nutrient elements [9], photosynthesis and pigment synthesis [10] and enzyme activity, as well as the accumulation of secondary metabolites [11]. One of the crucial harmful effects of salinity is oxidative impairment due to a marked buildup of reactive oxygen species (ROS) such as superoxide, hydrogen peroxide, and hydroxyl radical [12]. The ROS accrual causes the oxidation of proteins and lipids [13] and impedes regular cellular functioning due to the malfunctioning of crucial cellular organelles, including chloroplasts [14].

Plants have evolved various protective mechanisms to survive under environmental cues such as inducing accumulation of osmolytes, leading to increased water uptake, the maintenance of physiological processes [15], and the modulation of the antioxidant defense system for the fast elimination of ROS [16]. The antioxidant defense system competently removes overaccumulation of ROS by altering the activities of various vital enzymes, e.g., catalase (CAT), superoxide dismutase (SOD), ascorbate peroxidase (APX), glutathione peroxidase (GPX), glutathione reductase (GR), dehydroascorbate reductase (DHAR), and monodehydroascorbate reductase (MDHAR) [16,17]. Besides enzymatic antioxidant systems, nonenzymatic metabolites such as glutathione (GSH) and ascorbate (AsA) can also effectively scavenge the ROS [16].

Methylglyoxal (MG) is an extremely reactive substance leading to oxidative damage in plants [18]. However, plants have evolved a glyoxalase pathway, including glyoxalase I (Gly I) and glyoxalase II (Gly II) enzymes, that can effectively detoxify MG into nonhazardous substances [19]. This suggests the possibility that effective control of the glyoxalase pathway and antioxidant defense system could be vital in enhancing plant response to saline stress [20].

To alleviate the deleterious effects of numerous stresses, including salinity, one of the sustainable methods could be the exogenous application of plant metabolites to improve plant salt tolerance with no damage to the plants [21]. Among several defensive compounds naturally synthesized by plants, glutathione (GSH) appears to have substantial biostimulatory potential in plant development that cannot be ensured by other antioxidants or thiols [22]. One of the functions of GSH is to play a positive role in reducing oxidative stress [23]. Under stress conditions, including salt stress, GSH has been reported to have a positive effect on plants [24,25]. GSH is consumed as a substrate in the glyoxalase system to detoxify MG [26] and it also maintains cellular redox status, stress signal transmission, and gene expression [27].

Some studies have depicted an effective role of externally applied GSH in counteracting a variety of stresses, such as heat stress in mung bean [28], Pb stress in wheat [29], and salt stress in mung bean [30], tomato [25,31], and soybean [24].

l-cysteine desulfhydrase (l-DES) is one of the key enzymes involved in hydrogen sulfide (H_2_S) synthesis [32,33]. This enzyme is mainly located in the cytoplasm, wherein it converts l-cysteine to pyruvate by releasing H_2_S and NH_4_^+^ [34]. Salt stress has been shown to elevate l-DES activity in alfalfa [35] and tobacco [36]. Several metabolic events in plants are well reported to be mediated by H_2_S in plants, so as to tolerate several types of stresses [37,38] such as cadmium stress [39], chromium stress [40], and salinity stress [41,42]. The principal aim of the study is to uncover the putative role of endogenous l-DES activity and H_2_S content in GSH-induced tolerance to salt stress in sweet pepper plants by using an inhibitor of l-DES, dl-propargylglycine (PAG), and a scavenger of H_2_S, hypotaurine (HT).

## 2. Materials and Methods

### 2.1. Plant Growth Conditions

An investigation in a greenhouse was set up with sweet pepper (*Capsicum annuum* L.) cv. “Semerkand”. The seeds were disinfected with NaOCl solution at 1% (*v/v*). Firstly, five seeds were planted in each plastic pot of 5-L capacity, filled with perlite. After germination, two seedlings were uprooted, and three seedlings in each pot were kept growing. The temperatures of the growth medium ranged from 20–25 °C to 10 °C, day and night, respectively. The photoperiod was 11 h during the growth period.

Details of Hoagland’s nutrient solution (NS) are highlighted by Kaya and Ashraf [43]; the NS’ pH was kept at 5.5. The NS was applied to the root growth medium of plants every other day during the experimentation.

**Figure 1:**
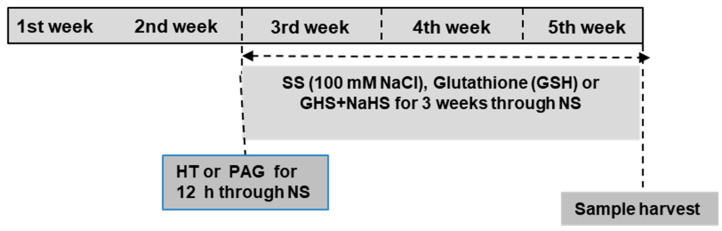
A scheme of the treatments used to study the effect of salinity stress (SS, 100 mmol L^−1^ NaCl) in sweet pepper plants. The concentrations used of each chemical were glutathione (1.0 mmol L^−1^ GSH) or sodium hydrosulfide (0.2 mmol L^−1^ NaHS) alone or combined with scavenger of H_2_S, hypotaurine (0.1 mmol L^−1^ HT), or inhibitor of l-DES, dl-propargylglycine (0.3 mmol L^−1^ PAG).

Figure 1 shows the scheme of the treatments used to assess the effect of salinity stress (SS) on sweet pepper plants. Two weeks after germination, plants were grown in NS without adding NaCl (control) or by adding 100 mmol L^−1^ NaCl (SS) for three weeks, and during the same period, glutathione (1.0 mmol L^−1^ GSH) or GSH+NaHS (1.0 mmol L^−1^ GSH + 0.2 mmol L^−1^ sodium hydrosulfide, H_2_S donor) was supplemented via nutrient solution. Furthermore, two weeks after germination, half of the young seedlings were retained for 12 h in an aqueous solution consisting of the H_2_S scavenger, hypotaurine (0.1 mmol L^−1^ HT), or l-DES inhibitor, dl-propargylglycine (0.3 mmol L^−1^ PAG).

The duration and concentrations of HT and PAG were selected according to the work of Li et al. [44] for maize plants exposed to high temperatures. A dose of 1 mmol L^−1^ GSH was used for mung bean [30] and soybean [45] under saline stress. These concentrations were selected for pepper plants in the present experiment.

Each treatment was replicated thrice, and each replicate comprised 9 plants, i.e., 27 plants for each treatment. Salinity stress of the root-growing medium was maintained constant throughout the experimentation by draining the excess amounts of NS through the plastic pots; this also prevented waterlogging.

Twenty-one days after initiating the salinity stress treatment, 3 plants from each replication were uprooted and separated into shoots and roots to measure dry mass. The separated plant parts were subjected to 75 °C in an oven for three days to determine dry mass. The remaining 6 plants from each replication were taken to determine the following parameters.

### 2.2. Photosynthetic Pigments and Chlorophyll Fluorescence

The protocol of Strain and Svec [46] was followed to quantify chlorophyll content. One g of fresh leaf tissue was macerated using acetone solution (5 mL, 90% *v/v*). The extracted sample solution was read at 663.5 and 645 nm for Chl *a* and Chl *b*, respectively.

For assessing the extent of chlorophyll fluorescence, leaf samples adapted at dark for 30 min were used. All fluorescence measurements were made with a photosynthesis yield analyzer (Mini-PAM, Walz, Germany).

### 2.3. Leaf Water Potential and Relative Water Content (RWC)

The protocol of Yamasaki and Dillenburg [47] was employed to determine RWC. Further details of the protocol are given by Kaya et al. [48].

For water-potential measurements, a fully expanded youngest leaf excised from each plant in the morning was subjected to a water-potential apparatus (a pressure chamber, PMS model 600, USA).

### 2.4. Proline Content

The procedure outlined by Bates et al. [49] was followed to determine leaf free proline.

### 2.5. Glycine Betaine (GB) Determination

GB content was determined, as described by Grieve and Grattan [50]. The initially dried leaf samples were ground to powder, and then, 0.5 g of the dried sample was extracted in 20 mL of deionized water by shaking at 25 °C for 24 h. The sample mixture was then treated with 2N H_2_SO_4_ (1:1). Then, 0.5-mL of the extract was added to 0.2 mL cold potassium iodide to start the reaction. The treated sample solution was centrifuged properly at 10,000× *g* for 15 min. The aliquot was added to 1,2-dichloroethane, and absorbance readings were recorded at 365 nm after 3 h. The standards of GB were prepared in 2N H_2_SO_4_ at a range of 50–200 μg mL^−1^.

### 2.6. Determination of l-Cysteine Desulfhydrase Activity (l-DES) and Hydrogen Sulfide (H_2_S) Content

The l-DES activity was determined by quantifying H_2_S release from l-cysteine in the presence of dithiothreitol (DTT) by employing the method of Li et al. [51]. A leaf tissue (5 g) was triturated with liquid N_2_, and the soluble protein was extracted in a 5-mL solution of Tris-HCl (20 mmol L^−1^, pH 8.0). With the objective of achieving the same amount of protein in each sample, an adjustment of the protein content in the treated sample solution was made at 100 µg mL^−1^. An aliquot (1 mL) of the extracted sample contained 0.8 mmol L^−1^ l-cysteine, 100 mmol L^−1^ Tris–HCl, 2.5 mmol L^−1^ DTT at pH 9.0, and a 10-µg protein solution. By adding l-cysteine to the sample solution, the reaction was initiated, and then the treated sample was incubated for 15 min at 37 °C. An aliquot (0.1 mL) of 30 mmol L^−1^ FeCl_3_ was treated with 1.2 mol L^−1^ HCl, and 0.1 mL of 20 mmol L^−1^ *N*, *N*-dimethyl-*p*-phenylenediamine dihydrochloride. Lastly, it was added to the extract. The optical density of the samples was quantified at 667 nm after leaving it at room temperature for 15 min.

Leaf H_2_S was quantified following the protocol outlined by Li et al. [51], which is based on the formation of methylene blue from dimethyl-*p*-phenylenediamine in H_2_SO_4_.

### 2.7. Quantification of H_2_O_2_ Levels

The Velikova et al. [52] protocol was pursued to determine the levels of leaf H_2_O_2_. A proportion of fresh leaf (0.5 g) was macerated in TCA (trichloroacetic acid, 3 mL of 1% *w/v*) and then 0.75 mL of aliquot was added to the potassium-phosphate buffer (0.75 mL of 10 mmol L^−1^, pH 7.0) and KI (1.5 mL of 1 mol L^−1^) solution. The OD of all treated samples was recorded at 390 nm.

### 2.8. Malondialdehyde (MDA) Assay

The leaf MDA was assayed per the procedure outlined by Heath and Packer [53]. A 100-mg fresh leaf was triturated in trichloroacetic acid (TCA, 0.5 mL 0.1% *w/v*), and then the sample extract was centrifuged for 10 min at 15,000× *g* at 4 °C. Thereafter, the aliquot was diluted using TCA (20%), and 0.5 mL of 1.5% (*w/v*) thiobarbituric acid (TBA) was added to the aliquot mixture, and then it was kept in a water bath at 95 °C for 25 min. The OD of all treated samples was noted at 532 nm after cooling down the final sample on ice.

### 2.9. Electrolyte Leakage (EL)

The method previously outlined by Dionisio-Sese and Tobita [54] was followed to measure EL.

### 2.10. Soluble Proteins and Antioxidant Enzymes

Briefly, 0.5 g of fresh leaf tissue was macerated in 50 mmol L^−1^ sodium-phosphate buffer, which consisted of 1% (*w/v*) soluble polyvinyl pyrrolidine. The homogenized solution was subjected to centrifugation at 20,000× *g* at 4 °C for 15 min. The catalase (CAT) activity was assayed as described by Kraus and Fletcher [55]. The peroxidase (POD) activity was determined as illustrated by Chance and Maehly [56], and that of SOD was determined by quantifying the SOD ability to inhibit the nitroblue tetrazolium (NBT) photochemical [57]. Total soluble proteins were quantified, as illustrated by Bradford [58].

### 2.11. Analysis of Ascorbate (AsA) and Glutathione (GSH)

A fresh leaf tissue (0.5 g) was used to quantify AsA and GSH contents. The samples were triturated using a cold mixture of metaphosphoric acid (3 mL of 5% *w/v*) and EDTA (1.0 mol L^−1^). Thereafter, the homogenized solution was subjected to centrifugation at 11,500× *g* for 12 min at 4 °C, and the supernatant was pipetted out for the determination of both GSH and AsA.

The quantification of AsA level was done per the procedure of Huang et al. [59]. Then, 0.4 mL of the sample aliquot was neutralized with 0.6 mL of 500 mmol L^−1^ potassium-phosphate buffer (pH 7.0), and then it was determined in 100 mmol L^−1^ potassium-phosphate buffer (at pH 7.0), including 0.5-unit ascorbate oxidase at 265 nm.

The quantification of glutathione disulfide (GSSG) and oxidized GSH was assayed, employing the procedure of Yu et al. [60]. An aliquot (0.4 mL) of the sample solution was added to 0.6 mL of the potassium-phosphate buffer (500 mmol L^−1^ at pH 7.0). Glutathione is estimated by the change in absorption rate at 412 nm for NTB (2-nitro-5-thiobenzoic acid), a product which results in the DTNB [5,5′-dithio-bis (2-nitrobenzoic acid)] reduction. The concentration of GSSG was appraised by abolishing GSH with a derivatizing agent, 2-vinylpyridine.

### 2.12. Plant Crude Extracts

A protocol described by Shan and Liang [61] was used to estimate the enzymes’ activities. Each single leaf sample (0.5 g) was ground to powder using liquid nitrogen, and then, the sample was added to a test tube to which 6 mL of ice-cold extract solution consisting of 50 mmol L^−1^ KH_2_PO4 (pH 7.5), 0.1 mmol L^−1^ ethylenediaminetetraacetic acid (EDTA), Triton X-100 (0.3% *v/v*), and soluble polyvinylpolypyrrolidone (1% *w/v*) was added. For the APX assay, 1 mmol L^−1^ AsA was also used. The sample mixtures were rapidly centrifuged for 15 min at 13,000× *g* at 2 °C, and then the following enzymes were assayed rapidly in this aliquot.

### 2.13. Enzyme Assay

A protocol described by Nakano and Asada [62] was adopted to quantify the APX activity by following a decrease in optical density at 290 nm. The sample mixture contained 50 mM phosphate buffer (pH 7.3), 1 mmol L^−1^ AsA, 0.1 mmol L^−1^ EDTA, 1 mmol L^−1^ H_2_O_2_, and the enzyme extract.

A protocol illustrated by Grace and Logan [63] was used to estimate the GR activity by following a decline in OD at 340 nm. A reaction solution was prepared, which comprised 100 mmol L^−1^ Tris-HCl (pH 8.0), 0.5 mmol L^−1^ EDTA, 0.5 mmol L^−1^ MgCl_2_, 10 mmol L^−1^ oxidized glutathione (GSSG), 1 mmol L^−1^ NADPH, and the enzyme extract.

A protocol illustrated by Miyake and Asada [64] was used to assay the activity of MDHAR (EC 1.6.5.4) by following a decrease in OD at 340 nm. The solution of assay comprised 50 mmol L^−1^ Hepes-KOH (pH 7.6), AsA oxidase (2.5 Units; EC 1.10.3.3), NADH (1.0 mmol L^−1^), AsA (2.5 mmol L^−1^), and the enzyme extract.

A protocol described by Dalton et al. [65] was pursued to assay the activity of DHAR (EC 1.8.5.1) by consequently noting a decrease in OD at 265 nm. The treated sample solution had 100 mmol L^−1^ Hepes–KOH (pH 7.0), 20 mmol L^−1^ GSH, and 2 mmol L^−1^ DHA.

Glyoxalase I (Gly I) activity was quantified employing the protocol outlined by Hasanuzzaman et al. [66]. A solution of 100 mmol L^−1^ potassium-phosphate buffer at pH 7.0, comprising 15 mmol L^−1^ magnesium sulfate, 1.7 mmol L^−1^ GSH, and 3.5 mmol L^−1^ MG, was reacted with the enzyme assay solution. Optical density was read at 240 nm. For the quantification of glyoxalase II (Gly II) activity, the protocol delineated by Hasanuzzaman et al. [66] was adopted. A solution containing 100 mmol L^−1^ Tris-HCl buffer (pH 7.2), 0.2 mmol L^−1^ DTNB, and 1.0 mmol L^−1^ *S*-d-lactoylglutathione (SLG) was poured into the enzyme assay solution. Then, the OD values were read at 240 nm and the Gly-II activity estimated.

### 2.14. Methylglyoxal (MG) Levels

After homogenizing 0.5 g of the leaf sample 1:1 (*w/v*) in 5% perchloric acid, the homogenized solution was subjected to centrifugation at 11,000× *g* and 4 ºC for 10 min. Charcoal was mixed in the supernatant to abolish the color, and then a saturated potassium carbonate solution was also used to neutralize it at 25 °C. The *N*-acetyl-l-cysteine and sodium dihydrogen phosphate were added to the treated sample solution to attain a final volume of 1 mL. After 10 min, the synthesis of *N*-α-acetyl-*S*-(1-hydroxy-2-oxo-prop-1-yl) cysteine was noted at 288 nm [67].

### 2.15. Quantification of Mineral Nutrients

The powdered dry leaf tissues were ashed by subjecting all samples to 550 °C in a muffle furnace. The ash so obtained was mixed in 5 mL of 2 mol L^−1^ hot HCl for the estimation of Na^+^ and K^+^ contents [68]. Sodium (Na^+^) and K^+^ in the samples were analyzed with an ICP-OES (PerkinElmer Optima 5300 DV). The conditions of ICP-OES were RF Power 1.35 kW, plasma gas flow rate 12.1 L min^−1^, sample solution uptake rate 1.5 mL min^−1^, carrier gas flow rate 0.5 mL min^−1^, nebulizer gas flow rate 0.85 L min^−1^, and wavelengths 766.490 and 588.021 for K and Na, respectively.

### 2.16. Statistical Treatment to Data of All Attributes

The data for various variables were analyzed following an analysis of variance. Mean data, along with respective standard errors of each variable, were presented as bar diagrams. The mean values were compared for significant differences with Duncan’s multiple range test at *p* ≤ 0.001 level of probability.

## 3. Results

### 3.1. Treatment of GSH Enhances Plant Growth

**Figure 2:**
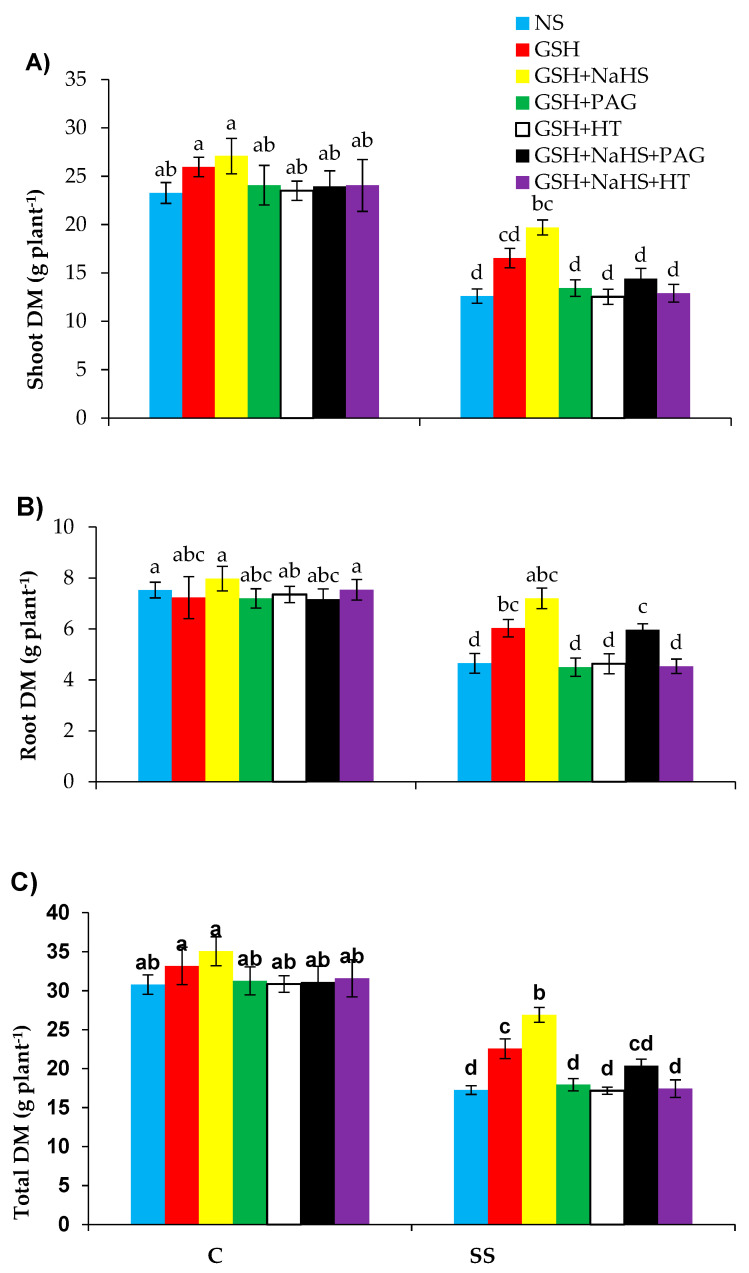
Shoot (**A**), root (**B**), total (**C**), and dry matter (DM) in pepper plants grown under control (C) and salinity stress (SS 100 mmol L^−1^ NaCl), sprayed with glutathione (1.0 mmol L^−1^ GSH) or sodium hydrosulfide (0.2 mmol L^−1^ NaHS) alone or together, combined with a 0.1 mmol L^−1^ scavenger of H_2_S, hypotaurine (0.1 mmol L^−1^ HT), or inhibitor of l-DES, dl-propargylglycine (0.3 mmol L^−1^ PAG). (Mean ± SE). Mean values carrying different letters within each parameter differ significantly (*p* ≤ 0.001) based on Duncan’s multiple range test.

Significant reductions (*p* ≤ 0.001) were obtained in shoot, root, and total dry weight of plants by 45.8%, 38.1%, and 43.9%, respectively, in the salt-stressed sweet pepper plants relative to the control. Decreases in these variables were significantly (*p* ≤ 0.001) raised by 31.2%, 29.4%, and 30.7%, respectively, with the application of GSH. Furthermore, increases in these variables were higher due to the treatment of GSH+NaHS relative to those in the salinity-stressed plants receiving no supplementation of GSH+NaHS (Figure 2A–C). The supplementation of l-DES inhibitor PAG or HT, in combination with GSH, totally reversed the positive effects of GSH on the plant growth parameters in the SS-plants by reducing the l-DES activity and H_2_S synthesis (as shown in the latter section), which suggests that the l-DES activity and synthesis of H_2_S participate in GSH-induced increased plant growth under SS. Furthermore, although HT reversed the beneficial role of GSH+NaHS, PAG did not completely reverse these effects under saline conditions. However, these different treatments did not affect those attributes in the control plants, showing that GSH or GSH+NaHS was only effective under stress conditions.

### 3.2. GSH Improves Photosynthesis-Related Parameters

**Figure 3:**
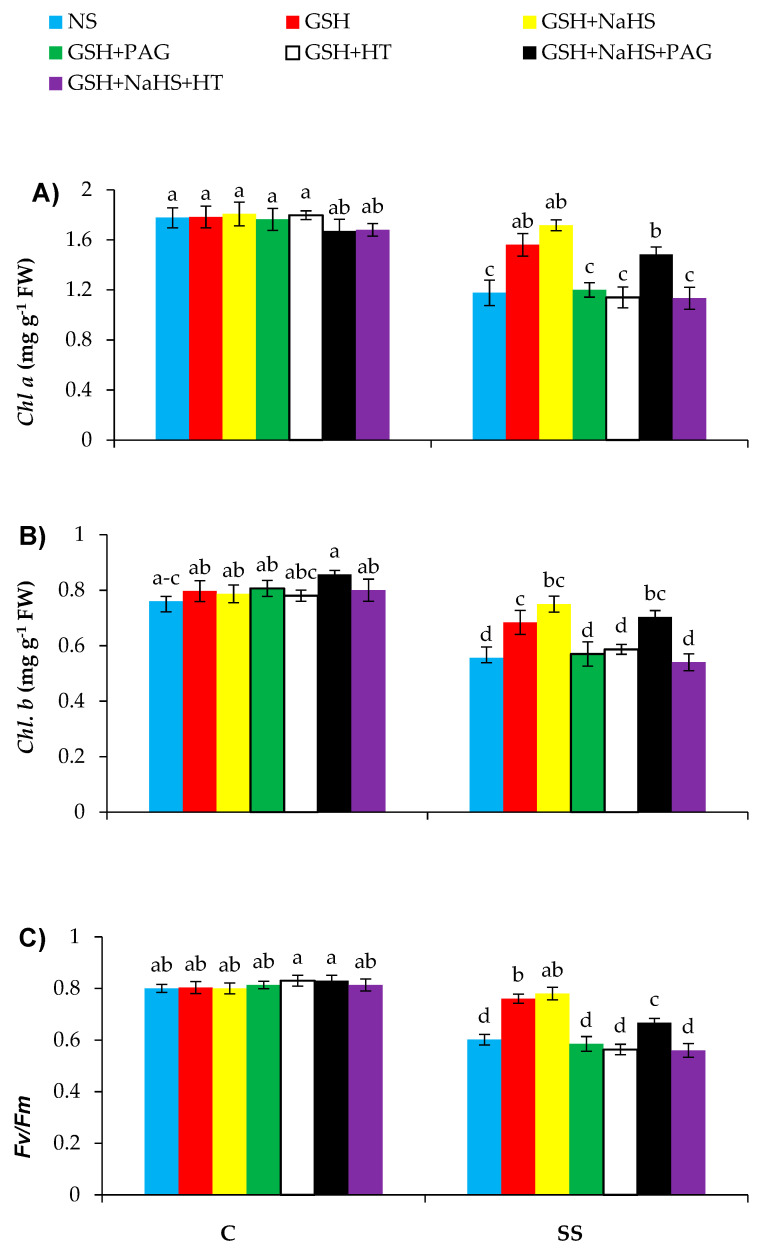
Chlorophyll *a* (**A**) and chlorophyll *b* (**B**) on a fresh weight (FW) basis and chlorophyll fluorescence parameters (*F_v_/F_m_* (**C**)) in pepper plants grown under control (C) and salinity stress (SS 100 mmol L^−1^ NaCl), sprayed with glutathione (1.0 mmol L^−1^ GSH) or sodium hydrosulfide (0.2 mmol L^−1^ NaHS) alone or together, combined with a 0.1 mmol L^−1^ scavenger of H_2_S, hypotaurine (0.1 mmol L^−1^ HT), or inhibitor of l-DES, dl-propargylglycine (0.3 mmol L^−1^ PAG). (Mean ± SE). Mean values carrying different letters within each parameter differ significantly (*p* ≤ 0.001) based on Duncan’s multiple range test.

Relative to the control plants, significant decreases, such as 33.4%, 26.8%, and 24.9% in Chl *a* and Chl *b* contents and photosystem II efficiency (*F_v_/F_m_*), respectively, were observed in the salt-stressed plants (Figure 3A–C). However, salt-stressed plants supplemented with GSH exhibited higher Chl *a*, Chl *b*, and *F_v_/F_m_* by 32.6%, 23.0%, and 26.4%, respectively, over those in the controls. Furthermore, the GSH+NaHS treatment led to a further increase in the photosynthesis-related parameter. Application of PAG and HT both reversed the mitigation effect of GSH on these attributes, showing that GSH-induced improvement in l-DES activity and H_2_S synthesis improves photosynthesis-related parameters under saline conditions. The mitigation effect of GSH+NaHS was completely reversed by the HT supply, thereby reducing the H_2_S content without inhibiting l-DES activity. However, this was partly reversed by PAG by completely blocking l-DES activity and partly reducing H_2_S, showing that H_2_S plays a vital role as a signal molecule in GSH-induced improvement in the photosynthesis-related attributes. However, all different treatments used did not alter those parameters in the control plants, showing that those treatments function only under stress conditions.

### 3.3. GSH Improves Leaf Water Relations and Proline and Glycine Betaine Contents

**Figure 4:**
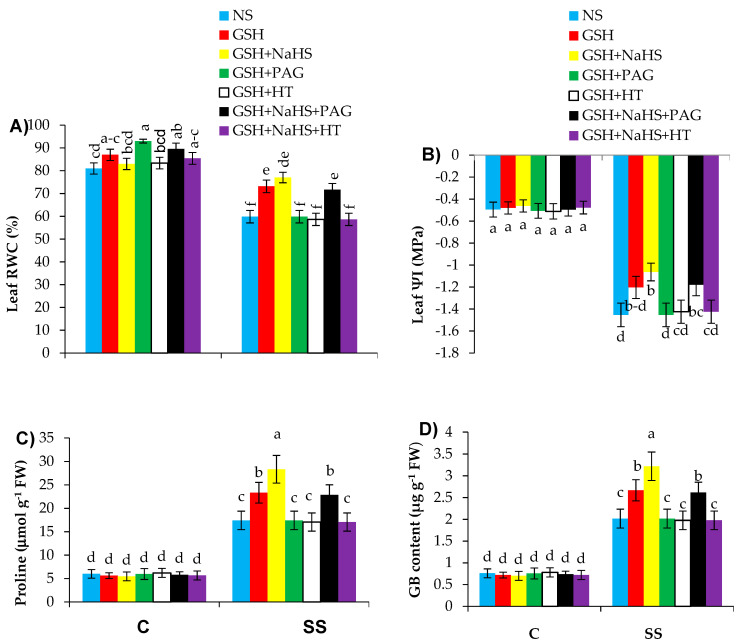
Leaf relative water content (RWC; (**A**)), leaf water potential (Ψl; (**B**)), proline (**C**), and glycine betaine (GB; (**D**)) contents in pepper plants grown under control (C) and salinity stress (SS 100 mmol L^−1^ NaCl), sprayed with glutathione (1.0 mmol L^−1^ GSH) or sodium hydrosulfide (0.2 mmol L^−1^ NaHS) alone or together, combined with a 0.1 mmol L^−1^ scavenger of H_2_S, hypotaurine (0.1 mmol L^−1^ HT), or inhibitor of l-DES, dl-propargylglycine (0.3 mmol L^−1^ PAG). (Mean ± SE). Mean values carrying different letters within each parameter differ significantly (*p* ≤ 0.001) based on Duncan’s multiple range test.

The water-related parameters, such as leaf relative water contents (RWC) and leaf water potential (ΨI), declined by 26.1% and 195.9%, respectively, in the salt-stressed plants over the controls. However, salt-stressed plants fed with GSH exhibited a lower reduction of 9.6% in RWC and 142.4% in ΨI relative to those in the controls (Figure 4A,B). The treatment of GSH+NaHS led to further increases in both RWC and ΨI of plants under SS. Salinity stress enhanced proline and glycine betaine (GB) contents by 2.9- and 2.6-fold, respectively, in the pepper plants, relative to those in the control treatment. The supplementation of the salt-stressed plants with GSH and GSH+NaHS led to a further rise in proline by 3.9- and 4.7-fold, and GB by 3.5- and 4.2-fold, respectively, over those in the control plants (Figure 4C,D). These attributes in the respective control plants remained unchanged under varying treatment regimes.

The beneficial effect of GSH on these attributes was totally inverted by PAG and HT by reducing both the activity of l-DES enzyme and H_2_S synthesis, showing that both l-DES enzyme and H_2_S synthesis are required in GSH-induced improvement in water status, and proline and GB contents under SS conditions. Furthermore, the alleviation effect of GSH+NaHS was completely prevented by HT by reducing NaHS without reducing the l-DES activity; it was partly prevented by PAG-induced reduction in the l-DES activity without completely reducing H_2_S.

### 3.4. GSH Improves l-DES Activity and H_2_S Synthesis in Pepper Plants under SS

**Figure 5:**
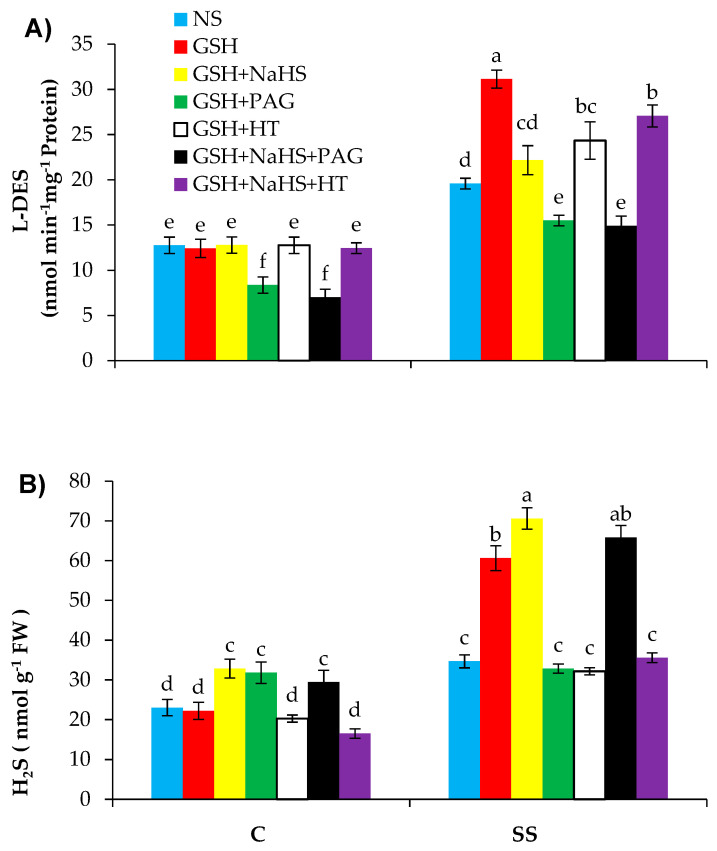
l-cysteine desulfhydrase (l-DES; (**A**)) and hydrogen sulfide (H_2_S (**B**)) in pepper plants grown under control (C) and salinity stress (SS 100 mmol L^−1^ NaCl), sprayed with glutathione (1.0 mmol L^−1^ GSH) or sodium hydrosulfide (0.2 mmol L^−1^ NaHS) alone or together, combined with a 0.1 mmol L^−1^ scavenger of H_2_S, hypotaurine (0.1 mmol L^−1^ HT), or inhibitor of l-DES, dl-propargylglycine (0.3 mmol L^−1^ PAG). (Mean ± SE). Mean values carrying different letters within each parameter differ significantly (*p* ≤ 0.001) based on Duncan’s multiple range test.

Salinity stress led to marked (*p* ≤ 0.001) increases in the activity of l-DES and synthesis of H_2_S by 53.5% and 50.4%, respectively, relative to those in the control treatment (Figure 5A,B). The supplementation of GSH resulted in a further significant (*p* ≤ 0.001) improvement in l-DES activity and led to a rise in the levels of endogenous H_2_S in the SS-pepper plants. These results indicate that GSH might have increased the accumulation of H_2_S by stimulating l-DES activity in the pepper seedlings under salinity stress. Application of GSH+NaHS further increased the H_2_S content but reduced the l-DES activity.

To assess the effect of the inhibitor of l-DES and the scavenger of H_2_S on GSH-induced improvement in salt tolerance in the sweet pepper plants, the plants were treated with GSH alone or jointly with NaHS, PAG, or HT. In the salt-stressed plants treated with GSH alone, the application of PAG totally prevented the increase in l-DES activity and H_2_S synthesis, but HT reversed the H_2_S content without reducing the l-DES activity. However, in the salt-stressed plants treated with GSH+NaHS, the PAG treatment totally reversed the l-DES activity without reducing the H_2_S content, and HT treatment prevented the increase in H_2_S content and l-DES activity. These results showed that PAG reduced the total l-DES activity, and in that way, it deactivated H_2_S synthesis, but HT directly scavenged H_2_S without hampering the l-DES activity. Furthermore, when NaHS was supplied externally along with GSH, PAG did not successfully reduce the H_2_S content. Our results also showed that H_2_S was produced by l-DES, which was activated by GSH under SS conditions.

### 3.5. GSH-Induced Rise in l-DES Activity and H_2_S Synthesis Relieve Oxidative Stress and Enhance Antioxidant Defence System in Pepper Plants under SS

**Figure 6:**
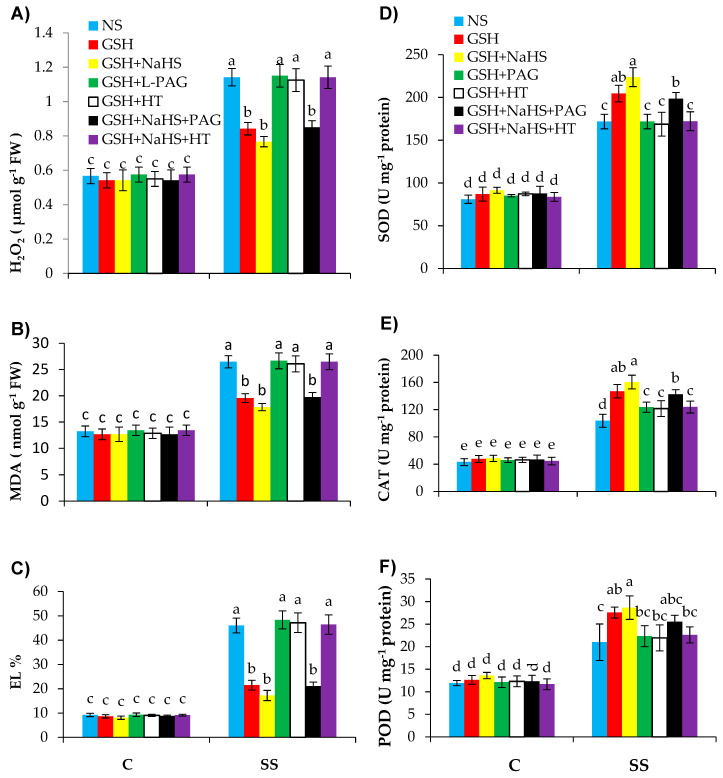
Hydrogen peroxide (H_2_O_2_; (**A**)), and malondialdehyde (MDA; (**B**)) on a fresh weight (FW) basis, and electrolyte leakage (EL; (**C**)) and activities of superoxide dismutase (SOD (**D**)), catalase (CAT (**E**)), and peroxidase (POD (**F**)) in pepper plants grown under control (C) and salinity stress (SS 100 mmol L^−1^ NaCl), sprayed with glutathione (1.0 mmol L^−1^ GSH) or sodium hydrosulfide (0.2 mmol L^−1^ NaHS) alone or together, combined with a 0.1 mmol L^−1^ scavenger of H_2_S, hypotaurine (0.1 mmol L^−1^ HT), or inhibitor of l-DES, dl-propargylglycine (0.3 mmol L^−1^ PAG). (Mean ± SE). Mean values carrying different letters within each parameter differ significantly (*p* ≤ 0.001) based on Duncan’s multiple range test.

The salt-stressed pepper plants exhibited a significant rise in oxidative stress-related parameters H_2_O_2_, MDA, and EL by 2.1-, 2.0-, and 4.9-fold, respectively, relative to those in the control plants (Figure 6A–C). Application of GSH to the salt-stressed plants reduced the accumulation of H_2_O_2_ and MDA and EL values by 30.6%, 26.2%, and 53.3%, respectively, over those in the SS-plants alone. Furthermore, the GSH and NaHS treatment further reduced the oxidative stress parameters, showing that the external application of NaHS along with GSH had a positive effect on reversing the oxidative stress. In the control plants, the oxidative stress-related parameters were not affected by various treatments.

However, PAG or HT treatment, along with GSH (GSH+PAG or GSH+HT), totally reversed the reduced oxidative stress due to GSH in the salt-stressed plants by reducing the H_2_S content and l-DES activity with GSH+PAG, and reducing H_2_S without reducing l-DES activity with the GSH+HT treatment. The reduced oxidative stress due to GSH+NaHS was not eliminated by PAG, even though PAG reduced the l-DES activity. However, the positive response in reducing oxidative stress due to the GSH+NaHS treatment was totally reversed by HT, thereby reducing the H_2_S content without reducing the l-DES activity.

Salinity stress significantly (*p* ≤ 0.001) increased the activities of antioxidant defense system-related enzymes, such as SOD, CAT, and POD by 2.1-, 2.9- and 1.9-fold, relative to those in the nonstressed plants (Figure 6D–F). The treatments of GSH and GSH+NaHS led to further increases in SOD by 19.2% and 30.4%, CAT by 15.6% and 29.8%, and POD by 17.1% and 26.1%, respectively, over those in the SS-plants, without being treated with GSH or GSH+NaHS. However, the upregulating effect of GSH on the antioxidant defense systems in the SS-plants was reversed by PAG or HT, thereby reducing the H_2_S content. The positive response of GSH+NaHS on the antioxidant defense system was not completely abolished by PAG, but was completely eliminated by HT, thereby reducing the H_2_S content.

### 3.6. The Participation of l-DES Activity and Endogenous H_2_S in GSH-Induced Regulation of Nonenzymatic Antioxidants in SS-Pepper Plants

**Figure 7:**
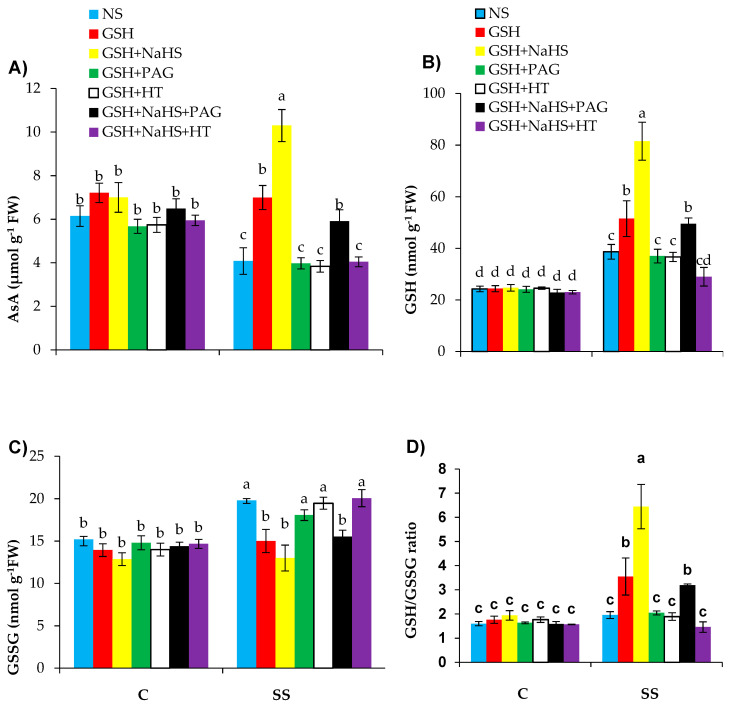
Ascorbate (AsA (**A**)), glutathione (GSH (**B**)), and glutathione disulfide (GSSG (**C**)) on a fresh weight (FW) basis, and GSH/GSSG ratio (**D**) in pepper plants grown under control (C) and salinity stress (SS 100 mmol L^−1^ NaCl), sprayed with glutathione (1.0 mmol L^−1^ GSH) or sodium hydrosulfide (0.2 mmol L^−1^ NaHS) alone or together, combined with a 0.1 mmol L^−1^ scavenger of H_2_S, hypotaurine (0.1 mmol L^−1^ HT), or inhibitor of l-DES, dl-propargylglycine (0.3 mmol L^−1^ PAG). (Mean ± SE). Mean values carrying different letters within each parameter differ significantly (*p* ≤ 0.001) based on Duncan’s multiple range test.

Plants subjected to SS showed a significant decrease in ascorbate (AsA) content by 33.4% but increases in the production of GSH and GSSG in the leaves of the plants under SS by 59.2% and 30.2%, respectively, relative to those under the control treatment (Figure 7A–C). The treatment of GSH led to significant increases in AsA and GSH contents by 71.5% and 112.8%, respectively, but decreases in GSSG content by 21.2% in the salt-stressed pepper compared to those in the plants treated only with salt. In the case of GSH+NaHS supplied to the SS-plants, significant increases were also observed in the AsA and GSH contents by 2.5-, and 2.2- fold, respectively; however, there were significant decreases in GSSG content by 34.1% in the SS-plants relative to those in the SS-plants receiving no supply of GSH+NaHS. The ratio of GSH/GSSG did not differ significantly under SS relative to the control. However, relative to SS alone, the exogenously applied GSH and GSH+NaHS significantly enhanced the GSH/GSSG ratio by 2.1- and 3.7-fold, respectively (Figure 7D).

PAG or HT supplemented to the SS-plants treated with GSH alone completely eliminated the positive effect of GSH by reducing AsA and GSH and lowering the GSSG levels, as well as reducing the GSH/GSSG ratio. Although the reversing effect of HT on the SS-plants treated with GSH+NaHS was similar to that of its effect on the plants treated with GSH alone, PAG did not show a complete reversing effect on AsA, GSH, and GSSG in the salt-stressed plants treated with GSH+NaHS.

### 3.7. The Participation of l-DES Activity and Endogenous H_2_S in GSH-Induced Upregulation of Ascorbate-Glutathione (AsA-GSH) Cycle in SS-Pepper Plants

**Figure 8:**
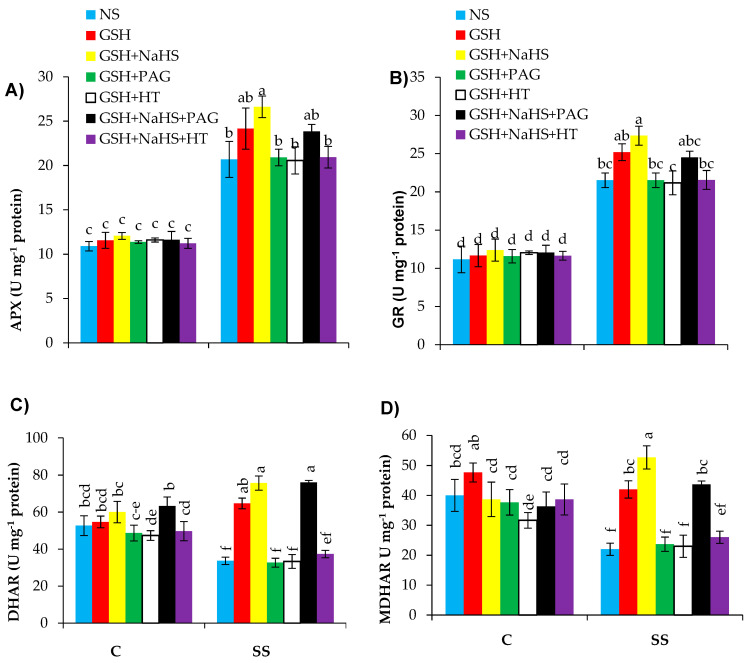
Activities of ascorbate peroxidase (APX (**A**)), glutathione reductase (GR (**B**)), dehydroascorbate reductase (DHAR (**C**)), and monodehydroascorbate reductase (MDHAR (**D**)) in pepper plants grown under control (C) and salinity stress (SS 100 mmol L^−1^ NaCl), sprayed with glutathione (1.0 mmol L^−1^ GSH) or sodium hydrosulfide (0.2 mmol L^−1^ NaHS) alone or together, combined with a 0.1 mmol L^−1^ scavenger of H_2_S, hypotaurine (0.1 mmol L^−1^ HT), or inhibitor of l-DES, dl-propargylglycine (0.3 mmol L^−1^ PAG). (Mean ± SE). Mean values carrying different letters within each parameter differ significantly (*p* ≤ 0.001) based on Duncan’s multiple range test.

To understand to what extent l-DES and H_2_S participate in SA-induced enhancement in salt stress tolerance by upregulating the AsA-GSH cycle in the pepper plants, alterations in the AsA-GSH-cycle-related enzymes’ activities were quantified in the leaves of the pepper seedlings. The salt-stressed plants showed enhanced activities of APX and GR by 91.65% and 90.1%, but reduced DHAR and MDHAR by 35.1% and 45.2%, respectively, over the controls (Figure 8A–D). The supply of GSH to the SS plants led to increased activities of APX, GR, DHAR, and MDHAR by 17.2%, 17.1%, 93.9%, and 90.9%, respectively, relative to those in the salt-stressed plants fed with no GSH. The treatment of GSH+NaHS led to further increases in these enzymes’ activities by 27.3%, 26.9%, 125.2%, and 139.4%, respectively, in comparison with those in the SS-plants alone.

Application of PAG or HT, along with GSH, completely prevented the alleviation effects of GSH on the previously mentioned traits in the SS-plants. This suggests that GSH might have activated the l-DES activity, which is the potential source of H_2_S, so H_2_S-induced upregulation of the AsA-GSH-cycle-related enzymes’ activities might have induced tolerance to SS in the pepper seedlings. When PAG or HT was applied along with the GSH+NaHS treatments, the upregulatory effects of GSH+NaHS on the AsA-GSH-cycle-related enzymes’ activities were completely reversed by HT, thereby reducing H_2_S without reducing the activity of l-DES; however, it was partly reversed by PAG, which reduced the accompanied l-DES activity by partly reducing H_2_S. This also suggests that H_2_S content is needed, but l-DES activity is not needed for the upregulation of the AsA-GSH-cycle-related enzymes due to GSH+NaHS under SS conditions, as long as the donor of H_2_S is supplied externally. These parameters remained unaltered in the control plants by different treatments.

### 3.8. GSH-Induced Enhanced l-DES Activity and H_2_S Synthesis Reduces Sodium and Improves Potassium and Calcium Contents in SS-Pepper Plants

**Figure 9:**
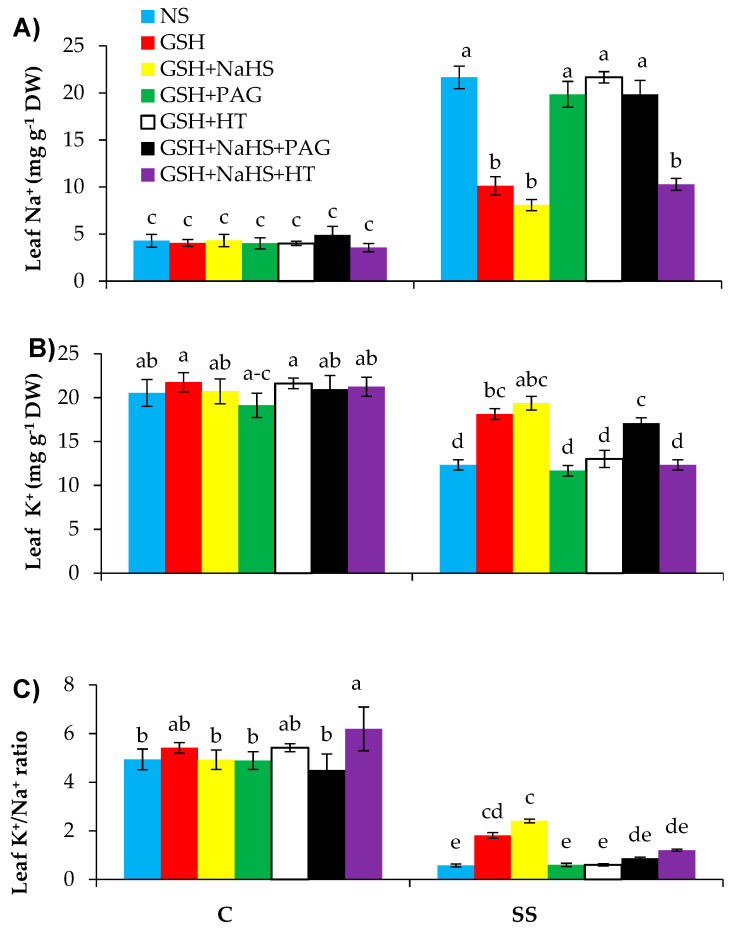
Leaf sodium (Na^+^ (**A**)), and potassium (K^+^ (**B**)) contents and leaf K^+^/Na^+^ ratio (**C**) in pepper plants grown under control (C) and salinity stress (SS 100 mmol L^−1^ NaCl), sprayed with glutathione (1.0 mmol L^−1^ GSH) or sodium hydrosulfide (0.2 mmol L^−1^ NaHS) alone or together, combined with a 0.1 mmol L^−1^ scavenger of H_2_S, hypotaurine (0.1 mmol L^−1^ HT), or inhibitor of l-DES, dl-propargylglycine (0.3 mmol L^−1^ PAG). (Mean ± SE). Mean values carrying different letters within each parameter differ significantly (*p* ≤ 0.001) based on Duncan’s multiple range test.

Saline stress caused a higher accumulation of Na^+^ in the leaves by 4.29-fold relative to the controls (Figure 9A). However, significant decreases were observed in the leaf K^+^ contents of the SS-plants by 41.9% over the controls (Figure 9B). The leaf K^+^/Na^+^ ratio (Figure 9C) was reduced in the salt-stressed plants by 7.4-fold over the controls.

The supplementation of GSH lowered the concentration of Na^+^ in the leaves by 50.9% in the SS-plants relative to those in the SS-plants alone. However, the same treatment led to increases in leaf K^+^ content and K^+^/Na^+^ ratio by 51.1% and 206.3%, respectively, in the plants treated with NaCl.

In order to provide further evidence that H_2_S is involved in GSH-induced SS tolerance in the pepper plants, NaHS (a donor of H_2_S) was supplied externally along with GSH (GSH+NaHS). The plants treated with GSH and NaHS in combination with GSH+NaHS led to further increases in the leaf K^+^ content and K^+^/Na^+^ ratio and a decrease in Na^+^ content. These results indicate that externally applied NaHS makes GSH more effective in improving the K^+^ content and lowering the Na^+^ content, and in this way, it increased the tolerance to SS in the pepper plants. In the salt-stressed pepper plants treated with GSH, the PAG or HT treatment completely reversed the decline in Na^+^ content and the rise in K^+^ content due to GSH, possibly by suppressing l-DES activity and H_2_S content or reducing the H_2_S content alone without reducing the l-DES activity. This provides evidence that endogenous H_2_S may be is involved in GSH-induced regulation of salt stress tolerance. Furthermore, for l-DES playing a key role in producing H_2_S signal molecules, the additional evidence proves that blocking the activity of l-DES by using its inhibitor leads to a decrease in H_2_S and reverses the positive effect of GSH. However, in the case of supplementing NaHS externally along with GSH, the PAG and HT treatments showed different effects in reversing the positive effect of GSH+NaHS. The treatment of HT reversed the positive effect of GSH+NaHS on K^+^ uptake by reducing the H_2_S content without reducing the l-DES enzyme activity. The PAG was less effective in reversing the positive effect of GSH+NaHS on K^+^ uptake, relative to the reversing effect of HT.

### 3.9. GSH-Induced l-DES Activity and H_2_S Synthesis Reduces Methylglyoxal Content and Upregulates Glyoxalase System Enzymes’ Activities in Pepper under SS

**Figure 10:**
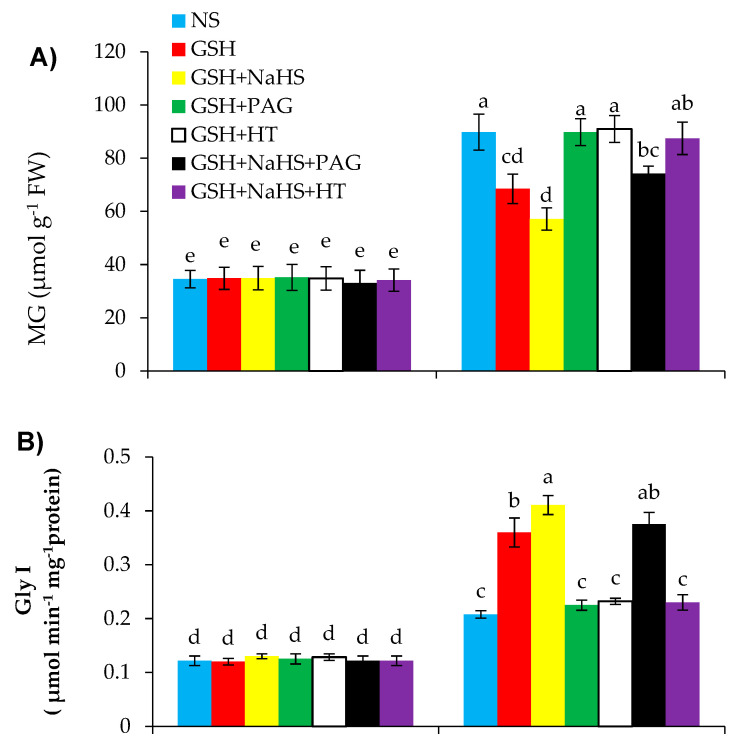

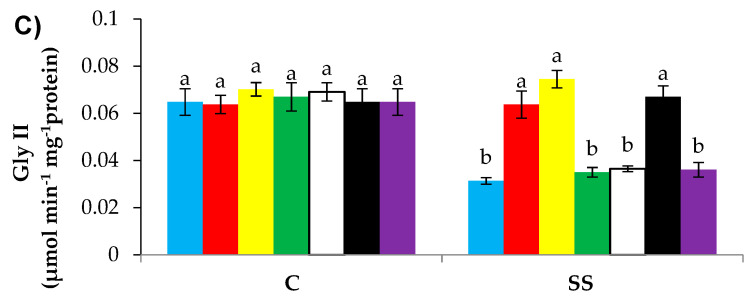
Methylglyoxal (MG (**A**)) content, glyoxalase I (Gly I (**B**)), and glyoxalase II (Gly II (**C**)) in pepper plants grown under control (C) and salinity stress (SS 100 mmol L^−1^ NaCl), sprayed with glutathione (1.0 mmol L^−1^ GSH) or sodium hydrosulfide (0.2 mmol L^−1^ NaHS) alone or together, combined with a 0.1 mmol L^−1^ scavenger of H_2_S, hypotaurine (0.1 mmol L^−1^ HT), or inhibitor of l-DES, dl-propargylglycine (0.3 mmol L^−1^ PAG). (Mean ± SE). Mean values carrying different letters within each parameter differ significantly (*p* ≤ 0.001) based on Duncan’s multiple range test.

Relative to the control plants, SS dramatically caused increased accumulation of methylglyoxal (MG) by 2.52-fold. However, the treatments of GSH and GSH+NaHS exhibited 23.7% and 36.3% less generation of MG, respectively, in the SS-plants over the controls (Figure 10A).

Salinity stress increased the activity of Gly I by 71.1%, but decreased that of Gly II by 51.1% over the controls (Figure 10B,C). Both GSH and GSH+NaHS supplemented to the SS-plants led to increases in the activity of Gly I by 73.4% and 98.1%, and that of Gly II by 103.1% and 137.2%, respectively relative to those in the SS-plants alone.

The positive effects of GSH on MG, Gly I, and Gly II were totally reversed by the supply of PAG and HT, as it was evidently observed that PAG or HT increased MG and reduced the activities of Gly I and Gly II. However, when NaHS was externally applied along with GSH (GSH+NaHS), HT eliminated the positive effect of GSH+NaHS on MG, Gly I, and Gly II by reducing the H_2_S content without significantly reducing the l-DES enzyme activity. PAG did not significantly reverse the positive effect of GSH+NaHS on the reduced content of MG; however, it increased the activities of both Gly I and Gly II, coupled with the reduced activity of l-DES.

### 3.10. SA-Induced Enhancement in Phenotypic Appearance of the Pepper Plants

**Figure 11:**
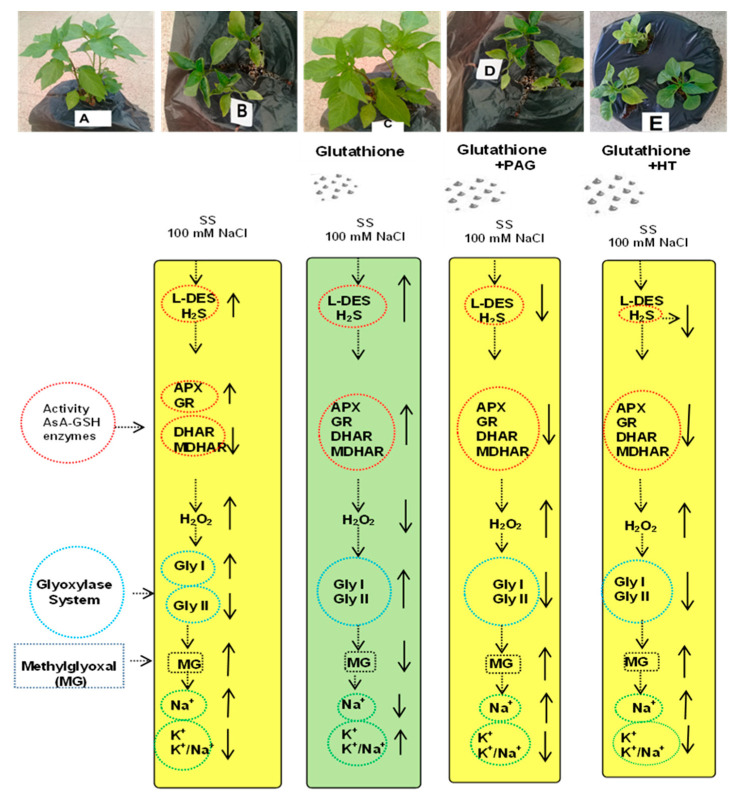
Effects of glutathione (GSH) and the inhibitor of l-cysteine desulfhydrase (l-DES), dl-propargylglycine (PAG), or the scavenger of hydrogen sulfide (H_2_S), hypotaurine (HT), on pepper plants subjected to control (**A**) and salinity stress (**B**), sprayed with glutathione (**C**) combined with inhibitor of l-DES, dl-propargylglycine (PAG; **D**) or scavenger of H_2_S, hypotaurine (HT; **E**). A proposed model depicting the participation of l-DES and H_2_S in GSH-induced SS tolerance in pepper plants.

As seen in Figure 11, the phenotypic appearance of the pepper plants showed that salt stress (SS) led to chlorosis, exhibiting yellow spots on the leaves. Salinity also caused reduced plant height and leaf size. Supplementation of GSH or GSH+NaHS eliminated such symptoms induced by SS. However, supplementation of the inhibitor of l-DES, PAG, or the scavenger of H_2_S, hypotaurine (HT), reversed the positive effect of GSH on the phenotypic appearance of plant leaves.

## 4. Discussion

Reduced plant growth is one of the well-known detrimental effects induced by salt stress [69,70,71], as has been consistently observed in this study. The reduced plant growth due to SS could be due to low uptake of essential nutrient elements [72], e.g., potassium (K^+^) [73] and calcium (Ca^+2^) [74], and Na^+^ toxicity [75]. An analogous observation has been obtained in the present study, wherein salinity stress reduced plant growth, leaf K^+^, followed by high Na^+^ accumulation. An alternatively assumed reason for reduced plant growth could be decreased water uptake in plants because of osmotic stress induced by salinity [76,77]. Similarly, leaf water content (RWC) and water potential were reduced in SS plants in the present study.

Our results indicated that GSH partly alleviated the deleterious effect of SS on plant growth, suggesting that GSH might be an active compound participating in SS tolerance of sweet pepper plants, as has previously been observed in rice [78] and tomato [25]. The positive response of GSH has previously been reported on some plant species under various stresses [27], such as water stress [79], high-temperature stress in mung bean [28], and lead stress in wheat [29]. The alleviation effect of GSH on salt-induced inhibited plant growth might be associated with enhancements in leaf chlorophyll content, *F_v_/F_m_*, and K^+^ content, which were initially depressed due to SS. Our results suggest that GSH can possibly contribute to the plant response to salt stress, as has formerly been observed in cotton [80] and tomato [81].

It has previously been reported that l-DES is known as the key enzyme for endogenous H_2_S generation in plants converting cysteine into H_2_S [82]. Inhibition in the activity of l-DES and endogenous H_2_S synthesis using PAG or HT reversed the positive effect of GSH under SS. This suggests that l-DES and H_2_S are both involved in GSH-induced SS tolerance in the pepper plants. Furthermore, our results reveal that GSH activates the l-DES enzyme, which is the main producer of H_2_S involved in improving tolerance to salt stress. Hence, H_2_S might act as a downstream signal molecule of l-DES that is activated by GSH. Furthermore, to provide further evidence that H_2_S plays a role in GSH-induced SS tolerance, NaHS was supplemented externally along with GSH. The application of GSH+NaHS led to a further increase in plant growth, showing that H_2_S could be involved in GSH-induced SS tolerance. The alleviation effect of GSH+NaHS was completely reversed by HT, but not by PAG, under saline conditions, suggesting that PAG did not successfully reverse the endogenous H_2_S content, although it blocked the activity of the l-DES enzyme when NaHS was supplied externally. These findings indicate that both the l-DES enzyme and H_2_S are needed for making the external application of GSH effective in regulating salinity tolerance. It has been well-reviewed that endogenous H_2_S plays a key role in salinity stress tolerance [42]. Externally supplemented H_2_S has been observed to alleviate the harmful effects of salt stress in several plant species, e.g., wheat [83], rice [84], and cucumber [42], as well as other stresses, such as cadmium stress in rice [85], drought stress in spinach [86], and chromium stress in maize [87]. However, no study has been reported in the available literature on the role of l-DES activity and H_2_S in GSH-induced SS tolerance. Hereafter, this could be the first report uncovering the mechanism of GSH-induced tolerance to SS.

The detrimental effect of salt stress on chlorophyll content has been widely observed in common bean [88] and cucumber [89], as similarly observed in our study. The decrease in chlorophyll due to SS in the pepper plants might have been associated with the explicit appearance of oxidative stress due to SS [88]. Furthermore, chlorophyllase enzyme might have been involved in blocking or deprivation of chlorophyll synthesis [90]. Another photosynthetic parameter affected by salinity is the *F_v_/F_m_*, which was reduced in SS-plants, as previously observed in various plants, e.g., in alfalfa [91], parsley [92], and cucumber [93]. Reduced *F_v_/F_m_* due to salinity showed that the reaction center of PSII is impaired or deactivated, resulting in photoinhibition in the stressed plants [92]. The existence of oxidative damage due to SS was related to reduced *F_v_/F_m_* [94].

The GSH-induced improvement in photosynthetic traits and reduction in H_2_O_2_ levels in the SS-plants reveal that GS participates in relieving the negative effects of SS on photosynthetic traits. These findings are consistent with those for other plant species, e.g., tomato [95] and mung bean [30]. GSH has also been shown to improve the synthesis of chlorophyll in salt-stressed rice plants [69].

It has been shown that GSH had a positive effect on endogenous l-DES activity and H_2_S synthesis in the salt-stressed pepper plants. However, pretreatment of PAG or HT disrupted the improvement in l-DES activity and H_2_S synthesis, and so both these pretreatments eliminated the improvement in chlorophyll content and *F_v_/F_m_*, indicating that both l-DES and H_2_S might have played a key function in GSH-enhanced chlorophyll content and *F_v_/F_m_*. Furthermore, the findings so obtained advocate that the role of the l-DES enzyme in GSH-induced improvement in salt tolerance might be due to the generation of H_2_S, which in turn participates in improving photosynthesis-related attributes. Analogous results have been obtained by other studies, exhibiting that externally applied H_2_S improved chlorophyll content in spinach under drought stress [86] and cucumber under salt stress [42].

The reduced leaf water status and increased proline and glycine betaine (GB) contents due to SS have been reported in various plants [96,97], similar to what has been observed in our study. The accumulation of proline under SS is known to be a mechanism responsible for stress tolerance of plants [98]. Glycine betaine is another osmoregulator that is actively involved in upregulating enzymes’ activities and, in turn, sustaining the integrity of membranes against the harmful effects of SS [99]. Although both proline and GB were found to be elevated in the pepper plants under SS, their accumulation might not have been enough to alleviate the oxidative stress due to the overgeneration of H_2_O_2_ in the present study. However, GSH led to further increases in proline and GB contents in the salt-stressed pepper plants. Herein, proline and GB accumulation could be crucial osmolytes in GSH-induced improvement in the leaf water status of plants under SS, as has been previously observed in mung bean by Nahar et al. [30].

The supplementation of PAG or HT reversed the beneficial effects of GSH on leaf water status and proline and GB under SS conditions, particularly by reducing the activity of l-DES and endogenous H_2_S content. This shows that both l-DES and H_2_S participate in GSH-induced improvement in leaf water status and proline and GB contents under SS. Furthermore, HT eliminated the positive effect of GSH+NAHS, but the reversing effect of PAG remained partial on the same parameters under SS. The previous reports show that exogenously applied H_2_S enhanced leaf water content in strawberry [100] and proline in rice [84] under salinity stress. These results show that endogenous H_2_S content is primarily needed in GSH-induced regulation of leaf water status and osmolytes (proline and GB), and l-DES is only needed to produce H_2_S when H_2_S is not supplied externally as NaHS.

In this study, l-DES activity and H_2_S synthesis increased in the SS-pepper plants, as have been reported earlier; salinity stress increased l-DES activity in alfalfa and tobacco [35,36]. Furthermore, l-DES activity and H_2_S synthesis were found to be further enhanced in the SS-plants treated with GSH and GSH+NaHS, showing that H_2_S acts as a signal molecule produced by l-DES, which is activated by GSH under SS conditions. This clearly shows that inhibition of l-DES activity by applying PAG causes a decrease in H_2_S synthesis/accumulation. The low concentration of H_2_S is thought to enhance the tolerance to abiotic and biotic stresses in plants, but high concentration can be detrimental to plant growth [101]. Hence, an acceptable level of H_2_S must be sustained in the plant cell to enhance the tolerance of plants to stress conditions. Therefore, in the current study, H_2_S induced by GSH was at an optimum level, which was not toxic to plants, providing evidence that no damaging effects on plant physiological processes were observed. Our results suggest that GSH might have activated the l-DES enzyme to synthesize H_2_S, but the beneficial effect of GSH was eliminated by supplementing PAG or HT through the reduction of l-DES activity and H_2_S content. So, the positive effect of GSH under SS could be due to the activation of H_2_S synthesis via increasing the activity of l-DES.

Salinity stress increased oxidative-stress-related parameters such as H_2_O_2_, MDA, and EL. This might have been due to the repressed activities of MDAR and DHAR, which are potential scavenger enzymes of H_2_O_2_, as similarly suggested by Yan et al. [81] under SS. The present observations also suggest that externally applied GSH is effective in inducing ROS detoxification in SS-pepper plants, and this could be one of the main strategies for GSH to relieve oxidative stress due to salinity and improve salinity tolerance. Such consistent observations were also reported in mung bean [30] and tomato [81]. Furthermore, NaHS applied along with GSH (GSH+NaHS) led to further reversal effects on oxidative stress parameters, showing that these two metabolites together have a stimulating effect on the reversal of oxidative stress. The pretreatment with PAG or HT had an eliminating effect on GSH-induced reduced oxidative stress under SS. This suggests that GSH might have triggered the l-DES activity, the potential source of H_2_S, and so H_2_S alleviating oxidative stress might have improved SS tolerance in the pepper plants. However, when GSH and NaHS were applied together (GSH+NaHS), the supply of HT completely reversed the positive effects of GSH+NaHS on oxidative stress, but that of PAG did not show a complete reversal effect. The reason for this could be that PAG did not eliminate the endogenous H_2_S, although it reversed the l-DES activity. In view of these data, it can be suggested that GSH-induced l-DES activity reverses oxidative stress under SS. However, for the maintenance of endogenous H_2_S at desirable levels by the supplementation of NaHS, GSH is not dependent on l-DES activity to reverse oxidative stress. The beneficial effect of H_2_S has previously been observed in different plant species, e.g., in maize [102], rice [84], and pepper [103].

The SOD is one of the antioxidant enzymes scavenging ROS by converting superoxide O_2_•− to H_2_O_2_ at the first stage of the defense system [104]. Salinity stress increased the SOD activity, and such consistent results were also observed in mung bean [30], chickpea [105], and soybean [21]. Application of GSH led to a further increase in the activity of SOD, as similarly observed in mung bean [30]. More stimulating effects on the SOD activity was obtained by providing NaHS and GSH jointly (GSH+NaHS).

Salinity stress increased the activities of CAT and POD, as has been observed in wheat [106]. The treatment of GSH further increased the activities of CAT and POD under SS, as has been observed in canola [107], mung bean [30], and tomato [81]. However, the supply of PAG or HT eliminated the positive effect of GSH on SOD, CAT, and POD activities under SS, suggesting that GSH-induced enhanced activities of the antioxidant enzymes is dependent on the l-DES activity and endogenous H_2_S accumulation. Externally supplied NaHS, a donor of H_2_S, has been shown to improve the activities of antioxidant enzymes under saline stress [106,108]. Pretreatment with HT eliminated the beneficial effect of GSH on SOD, CAT, and POD activities under SS, but PAG did not reverse the action of GSH when GSH was supplied along with NaHS (GSH+NaHS).

Ascorbate acts as a scavenger of ROS by either directly scavenging ROS or helping the detoxification of H_2_O_2_ via the AsA-GSH cycle [20,109]. Often, high AsA accumulation in plants helps plants to have a high tolerance to stress conditions [110]. In the present experiment, SS significantly decreased the AsA content due to the overaccumulation of H_2_O_2_ or the inhibition of the synthesis or restoring synthesis of AsA under stress conditions [20]. However, GSH supplementation led to increased AsA content in the SS- seedlings, perhaps by improving MDHAR and DHAR activities. These results were also consistent with the results reported in mung bean and *Brassica napus* under SS [20,30].

Glutathione has been reported to alleviate oxidative stress by eliminating ROS and is involved in maintaining the cellular redox status [20]. So, this special function of GSH makes it favorable to SS-plants [30]. The content of GSH increased in the SS-pepper plants, which is not consistent with some previous reports [30,111]. GSH contributes to ROS scavenging and is converted into GSSG, thereby increasing GSSG levels under SS, as observed in the present study. However, the addition of GSH reduced GSSG and increased GSH levels, as already observed in mung bean [30] and tomato [31] plants under saline stress. The ratio of GSH/GSSG is also one of the key indicators for the assessment of stress tolerance of plants. It means that the higher the GSH/GSSG ratio, the higher the stress tolerance of plants [112], as observed in this study with the exogenous application of GSH and GSH+NaHS. Salinity stress and addition of GSH and GSH+NaHS increased the activity of GR, which, in turn, helped to restore GSH and the GSH/GSSG ratio of the pepper plants to some extent. This has also been reported in mung bean plants under saline stress [30].

The supplementation of PAG and HT reversed the beneficial effect of GSH on nonenzymatic antioxidant levels; this was achieved by reducing the content of l-DES and H_2_S with PAG and by reducing H_2_S without lowering the l-DES activity with HT. However, PAG has not been fully successful in reversing the positive effect of GSH+NaHS when NaHS was supplied along with GSH (GSH+NaHS), since PAG reduced the activity of l-DES but did not reduce the H_2_S content completely. The HT pretreatment fully eliminated the positive effect of GSH+NaHS by inhibiting the H_2_S content without reducing l-DES activity. These results suggest that the l-DES activity and synthesis of H_2_S are involved in GSH-induced upregulation of nonenzymatic antioxidants under saline stress. However, when NaHS was supplied externally as a source of H_2_S, the positive effect of GSH did not depend on the l-DES activity. It has been shown that exogenously applied H_2_S upregulated AsA and GSH in maize [102] and wheat [113] plants under SS.

In the initial stage of the AsA-GSH cycle, APX scavenges H_2_O_2_ [114]. Previously, it has been described that APX can be positively modulated by H_2_S through persufidation [115]. Therefore, the increased activity of APX under saline stress is expected, as observed in this study. The supplementation of GSH and GSH+NaHS led to a further elevation in the activity of APX under SS, as consistently observed in mung bean and tomato plants under saline stress [30,81].

Another two enzymes related to the AsA-GSH cycle are the MDHAR and DHAR involved in AsA production [116]. In the present study, the SS-plants showed a decrease in MDHAR and DHAR activities, similar to what had been observed in *Brassica napus* by Hasanuzzaman et al. [20]. Nonetheless, the activities of DHAR and MDHAR were increased in the plants treated with GSH or GSH+NaHS, as consistently observed in tomato plants [107].

Glutathione reductase (GR) is also one of the key enzymes in the AsA-GSH cycle; it plays a crucial role in maintaining the pool or ratio of GSH/GSSG [98]. Furthermore, GR restores GSH from the oxidized GSSG using NADPH, which is generated predominantly from photosynthesis [27]. In this study, salt stress increased the activity of GR, as found in soybean and its activity was further elevated by the GSH and GSH+NaHS applications. Consistent results have also been reported in mung bean [30].

Pretreatment with PAG or HT eliminated the beneficial effect of GSH on the AsA-GSH-cycle-related enzymes by lowering H_2_S content. However, PAG has been partly effective in eliminating the positive effects of GSH+NaHS when NaHS was jointly supplied with GSH (GSH+NaHS). It is pertinent to note here that PAG did not completely reduce H_2_S content, but it completely reduced the activity of l-DES. The HT pretreatment fully eliminated the positive effect of GSH+NaHS by inhibiting the H_2_S content without reducing the l-DES activity. These data suggest that the l-DES activity and H_2_S play key functions in GSH-induced upregulation in the AsA-GSH-cycle-related enzymes under SS. Furthermore, the data show that the l-DES activity is not needed for making GSH, being effective for the upregulation of the AsA-GSH-cycle enzymes when NaHS was supplied externally as a source of H_2_S. The positive role of H_2_S on the AsA-GSH-cycle enzymes has previously been observed in plants under SS [102,113].

Plants require nutrient elements at an adequate rate to sustain their structure and key physiological processes [117]. However, these metabolic processes may be disturbed when such nutrients are not supplied sufficiently [118]. Salinity-induced high Na^+^ accumulation may occur due to potassium (K^+^) deficiency caused by the antagonistic effect of Na^+^ for K^+^ binding sites [119]. Therefore, an optimum K^+^/Na^+^ ratio is the main attribute for the salinity tolerance of plants [120]. Salinity stress lowers the K^+^/Na^+^ ratio because of the low level of K^+^ over that of Na^+^ in the leaves of plants [121]. However, GSH application lowered the leaf Na^+^ and elevated leaf K^+^ and the K^+^:Na^+^ ratio in the plants under saline stress, as recently reported by Zhou et al. [25].

Pretreatment with PAG or HT, along with GSH, eliminated the positive effect of GSH on leaf K^+^ and Na^+^ contents, as well as leaf K^+^/Na^+^ ratio, indicating that GSH promotes l-DES activity and H_2_S synthesis so as to enhance salt tolerance. The positive effect of externally applied GSH+NaHS was eliminated by HT by reducing the H_2_S content without reducing the l-DES activity, but it was not reversed by PAG, although PAG reduced the l-DES activity without reducing H_2_S. This shows that GSH-induced improvement in leaf K^+^ and the K^+^/Na^+^ ratio and reduction in Na^+^ contents is dependent on H_2_S content, but not on l-DES activity when NaHS is supplied externally as a source of H_2_S. Earlier studies have consistently shown that exogenously applied H_2_S improved salinity tolerance by reducing Na^+^ and increasing K and the K^+^/Na^+^ ratio in different plants, e.g., alfalfa [35], rice [84], and cucumber [40].

The accumulation of MG enhances in plant cells under stress conditions and it may be a potentially toxic compound that damages several physiological processes [30]. The plants subjected to SS showed high MG accumulation, as was observed in mung bean [30], *Brassica* spp. [122], and tomato [10] under SS. The glyoxalase system consists of two enzymes (Gly I and Gly II) to detoxify the overgeneration of methylglyoxal (MG) by consuming GSH as a substrate and thereby causes enhanced stress tolerance [123,124]. So, increased GSH content may impart a positive effect on reducing MG-induced oxidative stress [27]. In this study, increased GSH content under SS due to GSH treatment reduced the MG accumulation, thereby reducing oxidative stress in the pepper plants. Another strategy to reduce the MG accumulation is to upregulate the glyoxalase-system-related enzymes, Gly I and Gly II, in many plant species under stress conditions [122]. In the present study, SS increased the activity of Gly I, but decreased that of Gly II in the pepper plants, similar to what was observed in *Brassica napus* [20], tomato [125], and soybean [21]. Pretreatment with GSH significantly enhanced both enzyme activities and reduced the MG content, suggesting a detoxification effect of MG and improvement in SS tolerance in the pepper plants. Similar observations have also been reported in mung bean [30].

Pretreatment with PAG or HT eliminated the up-regulation effect of GSH on the glyoxalase system and positive effect of MG detoxification by inhibiting the activity of l-DES or synthesis of H_2_S in the pepper plants under SS. This shows that the beneficial effect of GSH is dependent on l-DES activity or H_2_S content. The beneficial effect of exogenously applied H_2_S in upregulating the glyoxalase system has been earlier reported in rice [78]. The benefit of externally applied GSH+NaHS was reversed by HT by lowering H_2_S without altering l-DES activity. This shows that GSH-induced improvement in the glyoxalase system and the reduction in MG accumulation need H_2_S alone rather than the l-DES activity when NaHS is supplied externally as a source of H_2_S.

## 5. Conclusions

As explained earlier, the suggested mechanism of GSH-induced SS tolerance in pepper plants is by increasing H_2_S due to the activation of the l-DES enzyme, which is the main producer of H_2_S. In this way, it alleviates the oxidative stress by stimulating the AsA-GSH-cycle-related enzymes and the glyoxalase system, as well as by detoxifying the MG content, which, in turn, reduces Na^+^ content and increases K^+^ content. Additional proof shows that HT or PAG eliminates SS tolerance by reducing either H_2_S content or l-DES activity, and downregulating the AsA-GSH cycle and glyoxalase system, as well as eliminating the GSH-induced decrease in oxidative stress and MG content, thereby again enhancing Na^+^ and reducing K^+^ content in plants.

Upcoming investigation in this area under field conditions might contribute to sustainable crop production, particularly in soils contaminated with high salinity. Furthermore, the participation of other signaling molecules and enzymes in the GSH-induced response of plants under salt stress and other stressors also needs to be investigated.

## References

[1] Serret M.D., Yousfi S., Vicente R., Piñero M.C., Otálora-Alcón G., del Amor F.M., Araus J.L. (2018). Interactive effects of CO_2_ concentration and water regime on stable isotope signatures, nitrogen assimilation and growth in sweet pepper. Front. Plant Sci..

[2] Kijne W. (2003). Working Towards Unlocking the Water Potential of Agriculture. http://agris.fao.org/agris-search/search.do?recordID=SO2005100464.

[3] Lee S.K.D. (2006). Hot pepper response to interactive effects of salinity and boron. Plant Soil Environ..

[4] Hussain S., Shaukat M., Ashraf M., Zhu C., Jin Q., Zhang J. (2019). Salinity Stress in Arid and Semi-Arid Climates: Effects and Management in Field Crops.

[5] Kaya C., Ashraf M. (2020). The endogenous l-cysteine desulfhydrase and hydrogen sulfide participate in supplemented phosphorus-induced tolerance to salinity stress in maize (Zea mays) plants. Turk. J. Bot..

[6] Kumar V., Khare T. (2015). Individual and additive effects of Na+ and Cl− ions on rice under salinity stress. Arch. Agron. Soil Sci..

[7] Gul M., Wakeel A., Steffens D., Lindberg S. (2019). Potassium-induced decrease in cytosolic Na+ alleviates deleterious effects of salt stress on wheat (*Triticum aestivum* L.). Plant Biol..

[8] Yang Y., Guo Y. (2018). Unraveling salt stress signalling in plants. J. Integr. Plant Biol..

[9] Imtiaz M., Rizwan M.S., Mushtaq M.A., Ashraf M., Shahzad S.M., Yousaf B., Tu S. (2016). Silicon occurrence, uptake, transport and mechanisms of heavy metals, minerals and salinity enhanced tolerance in plants with future prospects: A review. J. Environ. Manag..

[10] Parvin K., Hasanuzzaman M., Bhuyan M.H.M., Mohsin S.M., Fujita M. (2019). Quercetin mediated salt tolerance in tomato through the enhancement of plant antioxidant defense and glyoxalase systems. Plants.

[11] Ashraf M.A., Iqbal M., Rasheed R., Hussain I., Riaz M., Arif M.S., Ahmad P., Ahanger M.A., Alyemeni M.N. (2018). Environmental Stress and secondary metabolites in plants: An overview. Plant Metabolites and Regulation Under Environmental Stress.

[12] Nxele X., Klein A., Ndimba B.K. (2017). Drought and salinity stress alter ROS accumulation, water retention, and osmolyte content in sorghum plants. S. Afr. J. Bot..

[13] Saini P., Gani M., Kaur J.J., Godara L.C., Singh C., Chauhan S.S., Ghosh M.K., Zargar S., Zargar M. (2018). Reactive Oxygen Species (ROS): A Way to Stress Survival. Plants in Abiotic Stress-Mediated Sensing and Signaling in Plants: An Omics Perspective.

[14] Noctor G., Reichheld J.P., Foyer C.H., Jürgens G., Pimpl P. (2018). ROS-related redox regulation and signaling in plants. Seminars in Cell Developmental Biology.

[15] Rajasheker G., Jawahar G., Jalaja N., Kumar S.A., Kumari P.H., Punita D.L., Kishor P.B.K., Khan M.I.R., Ferrante A., Reddy P.S., Khan N.A. (2019). Role and Regulation of Osmolytes and ABA Interaction in Salt and Drought Stress Tolerance. Plant Signaling Molecules.

[16] Parvin K., Hasanuzzaman M., Bhuyan M.H.M., Nahar K., Mohsin S.M., Fujita M. (2019). Comparative physiological and biochemical changes in tomato (*Solanum lycopersicum* L.) under salt stress and recovery: Role of Antioxidant Defense and Glyoxalase Systems. Antioxidants.

[17] Duhan S., Kumari A., Lal M., Sheokand S., Duhan S., Kumari A., Lal M. (2019). Oxidative Stress and Antioxidant Defense Under Combined Waterlogging and Salinity Stresses in Reactive Oxygen, Nitrogen and Sulfur Species. Plants: Production, Metabolism, Signaling and Defense Mechanisms.

[18] Mostofa M.G., Ghosh A., Li Z.G., Siddiqui M.N., Fujita M., Tran L.S.P. (2018). Methylglyoxal—A signaling molecule in plant abiotic stress responses. Free Radic. Biol. Med..

[19] Gupta B.K., Sahoo K.K., Ghosh A., Tripathi A.K., Anwar K., Das P., Singla-Pareek S.L. (2018). Manipulation of glyoxalase pathway confers tolerance to multiple stresses in rice. Plant Cell Environ..

[20] Hasanuzzaman M., Nahar K., Rohman M.M., Anee T.I., Huang Y., Fujita M. (2018). Exogenous Silicon Protects Brassica napus Plants from Salinity-Induced Oxidative Stress Through the Modulation of AsA-GSH Pathway, Thiol-Dependent Antioxidant Enzymes and Glyoxalase Systems. Gesunde Pflanz..

[21] Alam P., Albalawi T.H., Altalayan F.H., Bakht M.A., Ahanger M.A., Raja V., Ahmad P. (2019). 24-Epibrassinolide (EBR) confers tolerance against NaCl stress in soybean plants by up-regulating antioxidant system, ascorbate-glutathione cycle, and glyoxalase system. Biomolecules.

[22] Noctor G., Mhamdi A., Chaouch S., Han Y.I., Neukermans J., Marquez-Garcia B.E.L.E.N., Foyer C.H. (2012). Glutathione in plants: An integrated overview. Plant Cell Environ..

[23] Diaz-Vivancos P., de Simone A., Kiddle G., Foyer C.H. (2015). Glutathione–linking cell proliferation to oxidative stress. Free Radic. Biol. Med..

[24] Akram S., Siddiqui M.N., Hussain B.N., Al Bari M.A., Mostofa M.G., Hossain M.A., Tran L.S.P. (2017). Exogenous glutathione modulates salinity tolerance of soybean [*Glycine max* (L.) Merrill] at reproductive stage. J. Plant Growth Regul..

[25] Zhou Y., Diao M., Chen X., Cui J., Pang S., Li Y., Hou C., Liu H.-Y. (2019). Application of exogenous glutathione confers salinity stress tolerance in tomato seedlings by modulating ions homeostasis and polyamine metabolism. Sci. Hortic..

[26] Ahanger M.A., Alyemeni M.N., Wijaya L., Alamri S.A., Alam P., Ashraf M., Ahmad P. (2018). Potential of exogenously sourced kinetin in protecting *Solanum lycopersicum* from NaCl-induced oxidative stress through up-regulation of the antioxidant system, ascorbate-glutathione cycle and glyoxalase system. PLoS ONE.

[27] Hasanuzzaman M., Nahar K., Anee T.I., Fujita M. (2017). Glutathione in plants: Biosynthesis and physiological role in environmental stress tolerance. Physiol. Mol. Biol. Plants.

[28] Nahar K., Hasanuzzaman M., Alam M.M., Fujita M. (2015). Exogenous glutathione confers high temperature stress tolerance in mung bean (*Vigna radiata* L.) by modulating antioxidant defense and methylglyoxal detoxification system. Environ. Exp. Bot..

[29] Hasanuzzaman M., Nahar K., Anee T.I., Fujita M. (2018). Exogenous glutathione attenuates lead-induced oxidative stress in wheat by improving antioxidant defense and physiological mechanisms. J. Plant Interact..

[30] Nahar K., Hasanuzzaman M., Alam M.M., Fujita M. (2015). Roles of exogenous glutathione in antioxidant defense system and methylglyoxal detoxification during salt stress in mung bean. Biol. Plant..

[31] Zhou Y., Wen Z., Zhang J., Chen X., Cui J., Xu W., Liu H.Y. (2017). Exogenous glutathione alleviates salt-induced oxidative stress in tomato seedlings by regulating glutathione metabolism, redox status, and the antioxidant system. Sci. Hortic..

[32] Romero L.C., García I., Gotor C. (2013). l-Cysteine Desulfhydrase 1 modulates the generation of the signaling molecule sulfide in plant cytosol. Plant Signal. Behav..

[33] Guo H., Zhou H., Zhang J., Guan W., Xu S., Shen W., Foyer C.H. (2017). l-cysteine desulfhydrase-related H_2_S production is involved in OsSE5-promoted ammonium tolerance in roots of Oryza sativa. Plant Cell Environ..

[34] Li Z.G., Cardenas E., Packer L. (2015). Analysis of some enzymes activities of hydrogen sulfide metabolism in plants. Methods in Enzymology.

[35] Lai D., Mao Y., Zhou H., Li F., Wu M., Zhang J., Xie Y. (2014). Endogenous hydrogen sulfide enhances salt tolerance by coupling the reestablishment of redox homeostasis and preventing salt-induced K^+^ loss in seedlings of *Medicago sativa*. Plant Sci..

[36] da-Silva C.J., Fontes E.P.B., Modolo L.V. (2017). Salinity-induced accumulation of endogenous H_2_S and NO is associated with modulation of the antioxidant and redox defense systems in *Nicotiana tabacum* L. cv. Havana. Plant Sci..

[37] Corpas F.J. (2019). Hydrogen sulfide: A new warrior against abiotic stress. Trends Plant Sci..

[38] Corpas F.J., Palma J.M. (2020). H2S signaling in plants and applications in agriculture. J. Adv. Res..

[39] Tian B., Qiao Z., Zhang L., Li H., Pei Y. (2016). Hydrogen sulfide and proline cooperate to alleviate cadmium stress in foxtail millet seedlings. Plant Physiol. Biochem..

[40] Ahmad R., Ali S., Rizwan M., Dawood M., Farid M., Hussain A., Ahmad P. (2019). Hydrogen sulfide alleviates chromium stress on cauliflower by restricting its uptake and enhancing antioxidative system. Physiol. Plant..

[41] da-Silva C.J., Modolo L.V. (2018). Hydrogen sulfide: A new endogenous player in an old mechanism of plant tolerance to high salinity. Acta Bot. Bras..

[42] Jiang J.L., Tian Y., Li L., Yu M., Hou R.P., Ren X.M. (2019). H_2_S alleviates salinity stress in cucumber by maintaining the Na+/K+ balance and regulating H_2_S metabolism and oxidative stress response. Front. Plant Sci..

[43] Kaya C., Ashraf M. (2015). Exogenous application of nitric oxide promotes growth and oxidative defense system in highly boron stressed tomato plants bearing fruit. Sci. Hortic..

[44] Li Z.G., Xie L.R., Li X.J. (2015). Hydrogen sulfide acts as a downstream signal molecule in salicylic acid-induced heat tolerance in maize (*Zea mays* L.) seedlings. J. Plant Physiol..

[45] Ardebili N.O., Saadatmand S., Niknam V., Khavari-Nejad R.A. (2014). The alleviating effects of selenium and salicylic acid in salinity exposed soybean. Acta Physiol. Plant..

[46] Strain H.H., Svec W.A., Vernon L.P., Seely G.R. (1966). Extraction separation estimation and isolation of the chlorophylls. The Chlorophylls.

[47] Yamasaki S., Dillenburg L.C. (1999). Measurements of leaf relative water content in Araucaria angustifolia. Rev. Bras. Fisiol. Veg..

[48] Kaya C., Tuna A.L., Ashraf M., Altunlu H. (2007). Improved salt tolerance of melon (Cucumis melo L) by the addition of proline and potassium nitrate. Environ. Exp. Bot..

[49] Bates L.S., Waldren R.P., Teare I.D. (1973). Rapid determination of free proline for water stress studies. Plant Soil.

[50] Grieve C.M., Grattan S.R. (1983). Rapid assay for determination of water-soluble quaternary ammonium compounds. Plant Soil.

[51] Li Z.G., Gong M., Liu P. (2012). Hydrogen sulfide is a mediator in H2O2-induced seed germination in *Jatropha curcas*. Acta Physiol. Plant..

[52] Velikova V., Yordanov I., Edreva A. (2000). Oxidative stress and some antioxidant system in acid rain treated bean plants: Protective role of exogenous polyamines. Plant Sci..

[53] Heath R.L., Packer L. (1968). Photoperoxidation in isolated chloroplasts. I. Kinetics and stoichiometry of fatty acid peroxidation. Arch. Biochem. Biophys..

[54] Dionisio-Sese M.L., Tobita S. (1998). Antioxidant responses of rice seedlings to salinity stress. Plant Sci..

[55] Kraus T.E., Fletcher R.A. (1994). Paclobutrazol protects wheat seedlings from heat andparaquat injury Is detoxification of active oxygen involved?. Plant Cell Physiol..

[56] Chance B., Maehly C. (1955). Assay of catalase and peroxidases. Method. Enzymol..

[57] Van Rossum M.W.P.C., Alberda M., Van Der Plas L.H.W. (1997). Role of oxidative damage in tulip bulb scale micropropagation. Plant Sci..

[58] Bradford M.M. (1976). A rapid and sensitive method for the quantitation of micro gram quantities of protein utilizing the principle of protein-dye binding. Anal. Biochem..

[59] Huang C., He W., Guo J., Chang X., Su P., Zhang L. (2005). Increased sensitivity to salt stress in ascorbate deficient Arabidopsis mutant. J. Exp. Bot..

[60] Yu C.W., Murphy T.M., Lin C.H. (2003). Hydrogen peroxide-induced chilling tolerance in mung beans mediated through ABA-independent glutathione accumulation. Funct. Plant Biol..

[61] Shan C., Liang Z. (2010). Jasmonic acid regulates ascorbate and glutathione metabolism in *Agropyron cristatum* leaves under water stress. Plant Sci..

[62] Nakano Y., Asada K. (1981). Hydrogen peroxide is scavenged by ascorbate specific peroxidase in spinach chloroplasts. Plant Cell. Physiol..

[63] Grace S.C., Logan B.A. (1996). Acclimation of foliar antioxidant systems to growth irradiance in three broad-leaved evergreen species. Plant Physiol..

[64] Miyake C., Asada K. (1992). Thylakoid-bound ascorbate peroxidase in spinach chloroplasts and photoreduction of its primary oxidation product monodehydroascorbate radicals in thylakoids. Plant Cell. Physiol..

[65] Dalton D.A., Russell S.A., Hanus F.J., Pascoe G.A., Evans H.J. (1986). Enzymatic reactions of ascorbate and glutathione that prevent peroxide damage in soybean root nodules. Proc. Natl. Acad. Sci. USA.

[66] Hasanuzzaman M., Nahar K., Alam M.M., Fujita M. (2014). Modulation of antioxidant machinery and the methylglyoxal detoxification system in selenium-supplemented *Brassica napus* seedlings confers tolerance to high temperature stress. Biol. Trace. Elem. Res..

[67] Wild R., Ooi L., Srikanth V., Münch G. (2012). A quick, convenient and economical method for the reliable determination of methylglyoxal in millimolar concentrations: The N-acetyll-cysteine assay. Anal. Bioanal. Chem..

[68] Chapman H.D., Pratt P.F. (1962). Methods analysis for Soils, plants and waters. Soil Sci..

[69] Teh C.Y., Mahmood M., Shaharuddin N.A., Ho C.L. (2015). In vitro rice shoot apices as simple model to study the effect of NaCl and the potential of exogenous proline and glutathione in mitigating salinity stress. Plant Growth Regul..

[70] Tuna A.L., Kaya C., Ashraf M. (2010). Potassium sulfate improves water deficit tolerance in melon plants grown under glasshouse conditions. J. Plant Nutr..

[71] Bolat I., Kaya C., Almaca A., Timucin S. (2006). Calcium sulfate improves salinity tolerance in rootstocks of plum. J. Plant Nutr..

[72] Yokaş I., Tuna A.L., Bürün B., Altunlu H., Altan F., Kaya C. (2008). Responses of the tomato (*Lycopersicon esculentum* Mill.) plant to exposure to different salt forms and rates. Turk. J. Agric. For..

[73] Ahmad P., Alyemeni M.N., Abass Ahanger M., Wijaya L., Alam P., Kumar A., Ashraf M. (2018). Upregulation of antioxidant and glyoxalase systems mitigates NaCl stress in *Brassica juncea* by supplementation of zinc and calcium. J. Plant Interact..

[74] Adhikari B., Dhungana S.K., Kim I.D., Shin D.H. (2019). Effect of foliar application of potassium fertilizers on soybean plants under salinity stress. J. Saudi Soc. Agric. Sci..

[75] Methenni K., Abdallah M.B., Nouairi I., Smaoui A., Zarrouk M., Youssef N.B. (2018). Salicylic acid and calcium pretreatments alleviate the toxic effect of salinity in the Oueslati olive variety. Sci. Hortic..

[76] Gandonou C.B., Prodjinoto H., Zanklan S.E.A., Wouyou A.D., Lutts S., Montcho D.H., Mensah A.C.E.G. (2018). Effects of salinity stress on growth in relation to gas exchanges parameters and water status in amaranth (*Amaranthus cruentus*). Int. J. Plant Physiol. Biochem..

[77] Kalteh M., Alipour Z.T., Ashraf S., Marashi Aliabadi M., Falah Nosratabadi A. (2018). Effect of silica nanoparticles on basil (*Ocimum basilicum*) under salinity stress. J. Chem. Health Risks.

[78] Hussain B.N., Akram S., Burritt D.J., Hossain M.A. (2016). Exogenous glutathione improves salinity stress tolerance in rice (*Oryza sativa* L.). Plant Gene Trait..

[79] Nahar K., Hasanuzzaman M., Alam M., Fujita M. (2015). Glutathione-induced drought stress tolerance in mung bean: Coordinated roles of the antioxidant defence and methylglyoxal detoxification systems. AoB Plants.

[80] Ibrahim W., Ahmed I.M., Chen X., Wu F. (2017). Genotype-dependent alleviation effects of exogenous GSH on salinity stress in cotton is related to improvement in chlorophyll content, photosynthetic performance, and leaf/root ultrastructure. Environ. Sci. Pollut. Res..

[81] Yan Z., Ming D., Cui J.X., Chen X.J., Wen Z.L., Zhang J.W., Liu H.Y. (2018). Exogenous GSH protects tomatoes against salt stress by modulating photosystem II efficiency, absorbed light allocation and H_2_O_2_-scavenging system in chloroplasts. J. Integr. Agric..

[82] Álvarez C., Calo L., Romero L.C., García I., Gotor C. (2010). An Oacetylserine (thiol) lyase homolog with l-cysteine desulfhydrase activity regulates cysteine homeostasis in Arabidopsis. Plant Physiol..

[83] Ye S.C., Hu L.Y., Hu K.D., Li Y.H., Yan H., Zhang X.Q., Zhang H. (2015). Hydrogen sulfide stimulates wheat grain germination and counteracts the effect of oxidative damage caused by salinity stress. Cereal Res. Commun..

[84] Mostofa M.G., Saegusa D., Fujita M., Tran L.S.P. (2015). Hydrogen sulfide regulates salt tolerance in rice by maintaining Na+/K+ balance, mineral homeostasis and oxidative metabolism under excessive salt stress. Front. Plant Sci..

[85] Mostofa M.G., Rahman A., Ansary M.M.U., Watanabe A., Fujita M., Tran L.S.P. (2015). Hydrogen sulfide modulates cadmium-induced physiological and biochemical responses to alleviate cadmium toxicity in rice. Sci. Rep..

[86] Chen J., Shang Y.T., Wang W.H., Chen X.Y., He E.M., Zheng H.L., Shangguan Z. (2016). Hydrogen sulfide-mediated polyamines and sugar changes are involved in hydrogen sulfide-induced drought tolerance in *Spinacia oleracea* seedlings. Front. Plant Sci..

[87] Kharbech O., Houmani H., Chaoui A., Corpas F.J. (2017). Alleviation of Cr (VI)-induced oxidative stress in maize (*Zea mays* L.) seedlings by NO and H_2_S donors through differential organ-dependent regulation of ROS and NADPH-recycling metabolisms. J. Plant Physiol..

[88] Taïbi K., Taïbi F., Abderrahim L.A., Ennajah A., Belkhodja M., Mulet J.M. (2016). Effect of salt stress on growth, chlorophyll content, lipid peroxidation and antioxidant defence systems in *Phaseolus vulgaris* L.. S. Afr. J. Bot..

[89] Wu Y., Jin X., Liao W., Hu L., Dawuda M.M., Zhao X., Yu J. (2018). 5-Aminolevulinic Acid (ALA) alleviated salinity stress in cucumber seedlings by enhancing chlorophyll synthesis pathway. Front. Plant Sci..

[90] Sharafi E., Dehestani A., Farmani J., Parizi A.P. (2017). Bioinformatics evaluation of plant chlorophyllase, the key enzyme in chlorophyll degradation. Appl. Food Biotechnol..

[91] Zhang J., Duan Z., Zhang D., Zhang J., Di H., Wu F., Wang Y. (2016). Co-transforming bar and CsLEA enhanced tolerance to drought and salt stress in transgenic alfalfa (*Medicago sativa* L.). Biochem. Biophys. Res. Commun..

[92] Habibi G. (2017). Selenium ameliorates salinity stress in *Petroselinum crispum* by modulation of photosynthesis and by reducing shoot Na accumulation. Russ. J. Plant Physiol..

[93] Estaji A., Kalaji H.M., Karimi H.R., Roosta H.R., Moosavi-Nezhad S.M. (2019). How glycine betaine induces tolerance of cucumber plants to salinity stress?. Photosynthetica.

[94] Azzabi G., Pinnola A., Betterle N., Bassi R., Alboresi A. (2012). Enhancement of non-photochemical quenching in the bryophyte Physcomitrella patens during acclimation to salt and osmotic stress. Plant Cell Physiol..

[95] Liu H.F., He X.L., Xiao C.Y., Cui J.X., Xu W., Liu H.Y. (2014). Effects of exogenous GSH on photosynthetic characteristics and expression of key enzyme genes of CO_2_ assimilation in leaves of tomato seedlings under NaCl stress. China J. App. Ecol..

[96] Bhuiyan M.S., Maynard G., Raman A., Hodgkins D., Mitchell D., Nicol H. (2016). Salt effects on proline and glycine betaine levels and photosynthetic performance in *Melilotus siculus*, *Tecticornia pergranulata* and *Thinopyrum ponticum* measured in simulated saline conditions. Funct. Plant Biol..

[97] Liu H., Song J., Dong L., Wang D., Zhang S., Liu J. (2017). Physiological responses of three soybean species (Glycine soja, G. gracilis, and G. max cv. Melrose) to salinity stress. J. Plant Res..

[98] Kaur H., Bhatla S.C. (2016). Melatonin and nitric oxide modulate glutathione content and glutathione reductase activity in sunflower seedling cotyledons accompanying salt stress. Nitric Oxide.

[99] Rady M.O., Semida W.M., El-Mageed T.A.A., Hemida K.A., Rady M.M. (2018). Up-regulation of antioxidative defense systems by glycine betaine foliar application in onion plants confer tolerance to salinity stress. Sci. Hortic..

[100] Christou A., Manganaris G.A., Papadopoulos I., Fotopoulos V. (2013). Hydrogen sulfide induces systemic tolerance to salinity and non-ionic osmotic stress in strawberry plants through modification of reactive species biosynthesis and transcriptional regulation of multiple defence pathways. J. Exp. Bot..

[101] Zhang P., Luo Q., Wang R., Xu J. (2017). Hydrogen sulfide toxicity inhibits primary root growth through the ROS-NO pathway. Sci. Rep..

[102] Shan C., Liu H., Zhao L., Wang X. (2014). Effects of exogenous hydrogen sulfide on the redox states of ascorbate and glutathione in maize leaves under salt stress. Biol. Plant..

[103] Kaya C., Higgs D., Ashraf M., Alyemeni M.N., Ahmad P. (2019). Integrative roles of nitric oxide and hydrogen sulfide in melatonin-induced tolerance of pepper (*Capsicum annuum* L.) plants to iron deficiency and salt stress alone or in combination. Physiol. Plant.

[104] Tanveer M., Shabala S., Kumar V., Wani S.H., Suprasanna P., Tran L.S.P. (2018). Salinity Stress Tolerance in Crops in Salinity Responses and Tolerance in Plants. Targeting Sensory, Transport and Signaling Mechanisms.

[105] Ahmad P., Abdel Latef A.A., Hashem A., Abd_Allah E.F., Gucel S., Tran L.-S.P. (2016). Nitric Oxide Mitigates Salt stress by regulating levels of osmolytes and antioxidant enzymes in chickpea. Front. Plant Sci..

[106] Ding H., Ma D., Huang X., Hou J., Wang C., Xie Y., Guo T. (2019). Exogenous hydrogen sulfide alleviates salt stress by improving antioxidant defenses and the salt overly sensitive pathway in wheat seedlings. Acta Physiol. Plant..

[107] Khattab H. (2007). Role of glutathione and polyadenylic acid on the oxidative defense systems of two different cultivars of canola seedlings grown under saline conditions. Aust. J. Basic Appl. Sci..

[108] Wang Y., Li L., Cui W., Xu S., Shen W., Wang R. (2012). Hydrogen sulfide enhances alfalfa (Medicago sativa) tolerance against salinity during seed germination by nitric oxide pathway. Plant Soil.

[109] Akram N.A., Shafiq F., Ashraf M. (2017). Ascorbic acid-a potential oxidant scavenger and its role in plant development and abiotic stress tolerance. Front. Plant Sci..

[110] Yactayo-Chang J.P., Acosta-Gamboa L.M., Nepal N., Lorence A., Hossain M.A., Munné-Bosch S., Burrit D.J., Diaz-Vivancos P., Fujita M., Lorence A. (2017). The Role of Plant High-Throughput Phenotyping in the Characterization of the Response of High Ascorbate Plants to Abiotic Stresses. Ascorbic Acid in Plant Growth, Development and Stress Tolerance.

[111] Iqbal N., Umar S., Per T.S., Khan N.A. (2017). Ethephon increases photosynthetic-nitrogen use efficiency, proline and antioxidant metabolism to alleviate decrease in photosynthesis under salinity stress in mustard. Plant Signal. Behav..

[112] Rahman A., Hossain M.S., Mahmud J.A., Nahar K., Hasanuzzaman M., Fujita M. (2016). Manganese-induced salt stress tolerance in rice seedlings: Regulation of ion homeostasis, antioxidant defense and glyoxalase systems. Physiol. Mol. Biol. Plant.

[113] Shan C., Zhang S., Zhou Y. (2017). Hydrogen sulfide is involved in the regulation of ascorbate-glutathione cycle by exogenous ABA in wheat seedling leaves under osmotic stress. Cereal Res. Commun..

[114] He F., Li H.G., Wang J.J., Su Y., Wang H.L., Feng C.H., Xia X. (2019). Pe STZ 1, a C2H2-type zinc finger transcription factor from Populus euphratica, enhances freezing tolerance through modulation of ROS scavenging by directly regulating Pe APX 2. Plant Biotechnol. J..

[115] Aroca Á., Serna A., Gotor C., Romero L.C. (2015). S-sulfhydration: A cysteine posttranslational modification in plant systems. Plant Physiol..

[116] Kim J.J., Kim Y.-S., Park S.I., Mok J.-E., Kim Y.-H., Park H.-M., Kim I.-S., Yoon H.-S. (2017). Cytosolic monodehydroascorbate reductase gene affects stress adaptation and grain yield under paddy field conditions in Oryza sativa L. japonica. Mol. Breed..

[117] Marschner H. (2011). Marschner’s Mineral Nutrition of Higher Plants.

[118] Liang B., Ma C., Zhang Z., Wei Z., Gao T., Zhao Q., Ma F., Li C. (2018). Long-term exogenous application of melatonin improves nutrient uptake fluxes in apple plants under moderate drought stress. Environ. Exp. Bot..

[119] Gul M., Wakeel A., Saqib M., Wahid A. (2016). Effect of NaCl-induced saline sodicity on the interpretation of soil potassium dynamics. Arch. Agron. Soil Sci..

[120] Hanin M., Ebel C., Ngom M., Laplaze L., Masmoudi K. (2016). New insights on plant salt tolerance mechanisms and their potential use for breeding. Front. Plant Sci..

[121] Assaha D.V., Ueda A., Saneoka H., Al-Yahyai R., Yaish M.W. (2017). The role of Na+ and K+ transporters in salt stress adaptation in glycophytes. Front. Physiol..

[122] Hossain M.S., Hasanuzzaman M., Hoque M.E., Huq H., Rohman M.M. (2016). Salinity and drought-induced methylglyoxal detoxification in *Brassica* spp. and purification of a high active glyoxalase I from tolerant genotype. Plant Omics.

[123] Hoque T.S., Hossain M.A., Mostofa M.G., Burritt D.J., Fujita M., Tran L.S.P. (2016). Methylglyoxal: An emerging signaling molecule in plant abiotic stress responses and tolerance. Front. Plant Sci..

[124] Sankaranarayanan S., Jamshed M., Kumar A., Skori L., Scandola S., Wang T., Samuel M.A. (2017). Glyoxalase goes green: The expanding roles of glyoxalase in plants. Int. J. Mol. Sci..

[125] Ahmad P., Abd_Allah E.F., Alyemeni M.N., Wijaya L., Alam P., Bhardwaj R., Siddique K.H. (2018). Exogenous application of calcium to 24-epibrassinosteroid pre-treated tomato seedlings mitigates NaCl toxicity by modifying ascorbate-glutathione cycle and secondary metabolites. Sci. Rep..

